# Evolutionarily conserved properties of CLCA proteins 1, 3 and 4, as revealed by phylogenetic and biochemical studies in avian homologues

**DOI:** 10.1371/journal.pone.0266937

**Published:** 2022-04-13

**Authors:** Florian Bartenschlager, Nikolai Klymiuk, Christoph Weise, Benno Kuropka, Achim D. Gruber, Lars Mundhenk

**Affiliations:** 1 Faculty of Veterinary Medicine, Department of Veterinary Pathology, Freie Universität Berlin, Berlin, Germany; 2 Large Animal Models in Cardiovascular Research, Internal Medical Department I, Technical University of Munich, Munich, Germany; 3 Center for Innovative Medical Models, Ludwig-Maximilians University Munich, Munich, Germany; 4 Institute of Chemistry and Biochemistry, Core Facility BioSupraMol, Freie Universität Berlin, Berlin, Germany; Ecole Normale Supérieure de Lyon, FRANCE

## Abstract

Species-specific diversities are particular features of mammalian chloride channel regulator, calcium activated (*CLCA*) genes. In contrast to four complex gene clusters in mammals, only two *CLCA* genes appear to exist in chickens. *CLCA2* is conserved in both, while only the galline *CLCA1* (*gCLCA1*) displays close genetic distance to mammalian clusters 1, 3 and 4. In this study, sequence analyses and biochemical characterizations revealed that gCLCA1 as a putative avian prototype shares common protein domains and processing features with all mammalian CLCA homologues. It has a transmembrane (TM) domain in the carboxy terminal region and its mRNA and protein were detected in the alimentary canal, where the protein was localized in the apical membrane of enterocytes, similar to CLCA4. Both mammals and birds seem to have at least one TM domain containing CLCA protein with complex glycosylation in the apical membrane of enterocytes. However, some characteristic features of mammalian CLCA1 and 3 including entire protein secretion and expression in cell types other than enterocytes seem to be dispensable for chicken. Phylogenetic analyses including twelve bird species revealed that avian CLCA1 and mammalian CLCA3 form clades separate from a major branch containing mammalian CLCA1 and 4. Overall, our data suggest that gCLCA1 and mammalian CLCA clusters 1, 3 and 4 stem from a common ancestor which underwent complex gene diversification in mammals but not in birds.

## Introduction

The ***c****h****l****oride channel regulator*, ***c****alcium* ***a****ctivated* (*CLCA*) gene family has been implicated in the pathophysiology of inflammatory airway diseases, such as asthma [1–3] or cystic fibrosis [4–6], tumor progression and metastasis [7–11]. In this context, different CLCA proteins have been linked to cellular functions including mucus processing [12,13], activation of airway macrophages [14–16], the modulation of the anion channel TMEM16A [17,18], cellular differentiation such as mucus cell metaplasia [1,19–21], cellular senescence and apoptosis [7,22–27], or as adhesion molecules for metastatic tumor cells [28,29]. Due to their proposed biomedical relevance, CLCA molecules have been studied in healthy and diseased humans [2,4,5,26,30–37] and mammalian model organisms including mouse [38–44], pig [45–49], horse [3,50], and cat [51,52].

The mammalian genome contains multiple *CLCA* gene copies, which can be classified into four distinct clusters based on genetic distances [51]. All *CLCA* genes are located in a single, conserved gene locus flanked by the two genes “*outer dense fibre of sperm tails 2-like*”(*ODF2L*) and “*SH3-domain GRB2-like endophilin B1*” (*SH3GLB1*). Clusters 1 and 2 are conserved in mammals, and each species carries only one functional *CLCA1* and *CLCA2* homologue. In contrast, clusters 3 and 4 are diverse, characterized by inactivation and duplication events in several mammalian species. In pigs, sheep, and dry-nose primates including humans, the *CLCA3* gene underwent independent mutational inactivation including several frameshift and nonsense mutations as well as the introduction of an additional exon [51]. In contrast to this pseudogenization, the gene is duplicated in cattle and mice leading to two or three functional orthologues, respectively. In other species such as cats, cluster 3 consists of only a single functional orthologue. In cluster 4, the *CLCA4* gene is duplicated in pigs and mice with two or three representatives, respectively. In humans, cats, and cattle only one functional *CLCA4* gene exists in this cluster. Taken together, the *CLCA* gene family is characterized by conserved clusters, such as 1 and 2, and by diverse clusters, such as 3 and 4 in mammals [51].

This genetic heterogeneity is also reflected in the cellular expression patterns of the proteins. CLCA1 was detected in mucin-producing cells of different mammal species [3,30,49,50,52,53] and the CLCA2 proteins are commonly expressed in keratinocytes of stratified epithelia [27,47,52,54–56]. In contrast, the expression patterns of the cluster 3 proteins are diverse in mammalian species. A single intact CLCA3 has so far only been found and characterized to date in cats. It is expressed in mucus-producing submucosal cells, ciliated epithelial cells, and esophageal keratinocytes [51]. In species with duplicated and intact *CLCA3* genes such as mouse and cattle (*Bos taurus*), their gene products were localized to keratinocytes, secretory epithelial cells, smooth muscle cells, and vascular endothelial cells [38,40,42,57–61]. In mammals, the CLCA4 proteins are predominantly expressed in enterocytes [46,48,62]. The duplication products of CLCA4 may lead to different cellular niches. In pigs, for example, one was localized to enterocytes of the villus tips of the small intestine while the other duplicate was found in crypt epithelial cells of small and large intestine [46,48]. In humans and pigs and in contrast to mice, CLCA4 members are also expressed in the airways [33,46,63]. In cluster 3 and 4, the duplication events seem to broaden the tissue and cellular expression pattern of the CLCA proteins with a species-specific evolution. This duplication may be associated with a sub- or neofunctionalization, which is unknown to date.

Despite this diversity at the genomic level and in the expression pattern of the particular CLCA clusters in mammals, a common feature of their functional protein products is the entry into the secretory pathway and an autocatalytic posttranslational cleavage of the precursor protein into a larger amino (N)-terminal and a smaller carboxy (C)-terminal subunit [64]. The N-terminal subunit encompasses a cysteine-rich CLCA domain with a HExxH motif (PFAM identifier: pfam08434), a von Willebrand factor type A (vWA) domain (PFAM identifier: pfam13519) and a β-sheet rich domain [64]. The C-terminal cleavage fragment has a fibronectin type III domain (PFAM identifier: PF00041) [64]. Additionally, based on biochemical properties, the mammalian CLCA clusters can be divided into two groups: the proteins of cluster 1/3 are soluble proteins, which are secreted in their entirety by the cells [51,52,64–66], whereas the proteins of cluster 2/4 are anchored in the plasma membrane via a C-terminal transmembrane domain [47,62,64,67]. Only one study on the phylogenetic distribution of the zinc-binding amino-acid (aa) motif HExxH of the CLCA domain has been published so far [68]. Furthermore, no CLCA homologues of other vertebrates have been investigated in detail to date, which limits assertions about novel acquired and conserved traits of CLCA proteins in the mammal lineage. Recently, it was reported that the *CLCA* genes in chicken are flanked by *ODF2L* and *SH3GLB1* identically to mammals; however, only two homologues were found in this gene locus [51]. One galline homologue clustered with the conserved mammalian *CLCA2* genes. In contrast, the second *CLCA* homologue, *gCLCA1*, clustered not only with the mammalian cluster 1, but also with the diverse clusters 3 and 4.

Here, we test the hypothesis that gCLCA1 combines expressional and protein-biochemical characteristics of mammalian CLCA1, 3, and 4, which suggests a common ancestor with an independent expansion in the early mammalian lineage. We analyzed the genomic organization and protein structure of gCLCA1 *in silico*, characterized its protein-biochemical properties and expression patterns in comparison to mammalian CLCA1, 3, and 4. The data may help to elucidate the properties and putative functions of early ancestral members of this gene family and infer novel properties of the diverse CLCA clusters in mammals.

## Materials and methods

### Characterization of the *gCLCA* gene locus and phylogenetic analyses

In addition to the architecture of the g*CLCA* locus [51] detailed gene positions and sizes and exon-intron boundaries were extracted from the NCBI (https://www.ncbi.nlm.nih.gov/) database and Plog et al. [49]. In brief, CLCA protein sequences were obtained from the NCBI and ensembl (http://www.ensembl.org/index.html) databases and aligned. Phylogenetic trees were generated using the phylip package (Phylogeny Inference Package 3.6. J. Felsenstein. Department of Genome Sciences, University of Washington, Seattle, WA, USA). First, three independent trees were generated by the maximum likelihood (ml), most parsimony (mp), and 100 neighbor joining (nj) methods. The final tree was based on the ml tree, with branch nodes occurring also in mp. Mammalian and avian CLCA2 were used as an outgroup. For better identification of distinct evolutionary speed in different protein domains, separate trees were calculated for the N-CLCA domain which includes a catalytic domain (cat, corresponding to aa45-200 in chicken CLCA1) and a cysteine-rich domain (cys, aa201-289), the von Willebrand factor A domain (vWA, aa297-483), a β-sheet rich domain (bsr, aa484-698), and fibronectin type III domain (fn3, aa754-874).

### *In silico* amino-acid sequence analysis of gCLCA1 and generation of antibodies

The aa sequence of the gCLCA1 was analyzed via NCBI Conserved Domain Database [69], EMBL-EBI HMMER web server [70], SUPERFAMILY 2 database [71,72], Phobius webserver [73], SOSUI [74], CCTOP [75], and SignalP 5.0 [76] algorithms to identify putative protein domains. Asparagine (N)-linked glycosylation sites were predicted using NetNGlyc 1.0 (http://www.cbs.dtu.dk/services/NetNGlyc/). The putative proteolytic cleavage site was identified by interspecies comparison with aa sequences of mammalian CLCA proteins. Anti-gCLCA1 antibodies were generated as described [49]. In brief, according to immunogenicity predictions, two oligopeptides were synthesized corresponding to aa92 to 105 (KKNSTYSRLKTESY, gCN1) and aa821 to 834 (ASVPSDDEGNTSDG, gCC1). *Limulus polyphemus* hemocyanin-conjugated peptides were used for immunization of two rabbits each. Specific IgG-antibodies were purified from the antisera using a cyanogen bromide immunization peptide-coupled sepharose column. The immunopurified polyclonal antibodies generated against the N-terminal and C-terminal part of the gCLCA1 protein were named gC1-N1 and gC1-C1, respectively.

### Animals and tissue processing

44 tissues each (S1 File) from ten-week old female chickens (Hampshire x White Leghorn, n = 3) and the gonads of male chickens (Hampshire x White Leghorn, n = 3) of the same age were snap-frozen on dry ice subsequent to a brief immersion in 2-methylbutane. For immunohistology or immunofluorescence, tissues were fixed in 4% neutral-buffered formaldehyde for 48 h and embedded in paraffin (FFPE-tissues). All tissues were by-products from slaughtered animals intended for human consumption. The animals had been bred, housed, and slaughtered in the Albrecht Daniel Thaer-Institute of Agricultural and Horticultural Sciences of the Humboldt-Universität zu Berlin, Germany (State Office of Health and Social Affairs Berlin, approval number IC 114-ZH70). Weight at harvest was 1–1.2 kg (females) and 1.3–1.5 kg (males). The animals were raised on miscanthus litter in groups of 25, with infrared heat lamps offered until week five. The animals were fed with fledgling rearing feed until week eight and pullet feed afterwards. Females were harvested in the morning and males in the morning of the following day.

### Molecular cloning of *gCLCA1* and generation of *gCLCA1* mutants

*gCLCA1* was cloned and mutants were generated as described in detail in S2 File. In brief, the *gCLCA1* open reading frame (ORF) was amplified from reverse transcribed RNA extracted from the proctodeum and tagged with the enhanced yellow fluorescent protein (*EYFP*) at the C-terminus by cloning into the p*EYFP*-N1 vector (*gCLCA1WT*). For analysis of an EQ mutation in the zinc-binding aa motif in accordance with Pawlowski et al. [77] and Bothe et al. [78], the wild type motif was replaced by a synthesized gene fragment containing a substitution of glutamic acid (E) with glutamine (Q) at position 164 (*gCLCA1E164Q*). As none of the specific anti-gCLCA1 antibodies were applicable for immunoblotting (see Immunoblotting section), a *gCLCA1*-fragment was substituted by a synthesized gene fragment containing a recognition site for murine N-terminal CLCA1 antibodies (α-p3b2, [53]) in order to enable for the detection of the N-terminal cleavage product of the murinized wild type (*gCLCA1Nmabc1)* or murinized EQ mutant (*gCLCA1Nmabc1E164Q) in vitro*.

### Tissue expression pattern of *gCLCA1* mRNA

The *gCLCA1* mRNA organ-specific expression pattern was analyzed by RT-qPCR as described in detail in S2 File. In brief, total RNA was isolated from tissues (S1 File), reverse transcribed, and the synthesized cDNA was diluted to a final concentration of 1 ng/μl. Specific, exon-boundaries spanning primers were used to detect *gCLCA1* or the reference gene phosphoglycerate kinase 1(*PGK1*) [79] using a SYBR green qPCR assay. *gCLCA1-*mRNA was considered to be expressed, when C_t_-values of 35 or below were detected in at least two of three samples tested.

### Transient transfection of HEK293 cells

HEK293 cells were transfected as described in detail in S2 File. In brief, the cells were grown in six-well plates in Dulbecco’s Modified Eagle’s Medium (DMEM) supplemented with 10% heat-inactivated fetal calf serum (FCS), 1% HEPES, and 1% penicillin/streptomycin. When the cells were approximately 80–90% confluent, they were transfected using 2 μg of a plasmid (*EYFP*-mock, pcDNA3.1^+^-mock, *gCLCA1WT*, *gCLCA1E164Q*, *gCLCA1Nmabc1*, *gCLCA1Nmabc1E164Q*, *mCLCA1* [78], *mCLCA1E157Q* [78], *mCLCA4a* [80], or *mCLCA4aE157Q* [80]) and 8 μl polyethylenimine (PEI, 1 mg/ml) per well. 12 h after transfection, the cells were washed with phosphate buffered saline (PBS) and serum-free DMEM was added to the cells. 72 h after transfection, the cell culture medium was removed and centrifuged at 14000 x g for one h at 4°C. The supernatant was concentrated using Vivaspin 2 protein concentrator spin columns (Sartorius, Göttingen, Germany) with a 10 kDa molecular weight cutoff. The cells of each well were lysed using 500 μl radioimmunoprecipitation assay (RIPA) buffer supplemented with a protease inhibitor cocktail. The protein concentration of supernatants and cell lysates were quantified using the bicinchoninic acid (BCA) method prior to freezing at –20°C.

### Endoglycosidase treatment

For glycosylation analysis, lysates from *gCLCA1Nmabc1*-transfected cells were deglycosylated by incubation with 25 U/ml endo H, 50 U/ml PNGase F, or left untreated at 37°C overnight according to the manufacturer´s protocol (New England Biolabs, Ipswich, Massachusetts, USA) and consequently immunoblotted as described in the Immunoblotting section.

### Immunoblotting

Cell lysates and supernatants of transfected cells were analyzed by immunoblotting as described in detail in S2 File. In brief, samples of cell lysates or concentrated cell culture supernatant were reduced in 1,4-dithiothreitol (DTT) and separated in a 10% acrylamide gel. The proteins were transferred onto a polyvinylidene fluoride (PVDF)-membrane and blocked with 5% non-fat milk. The membranes were probed with gC1-N1 and gC1-C1 both at a three-fold dilution series from 5 μg/ml to 0.05 μg/ml, or mouse monoclonal anti-YFP (cat. G163, abm, Vancouver, Canada) at 1:500, or rabbit polyclonal anti-mCLCA1 [53] at 1:500, or rabbit polyclonal anti-mCLCA4a [62], or mouse monoclonal anti beta-actin (A5441, MilliporeSigma, Saint Louis, Missouri, USA) antibodies at 1:1000. The membranes were incubated with horseradish peroxidase-conjugated goat anti-rabbit (115-035-068, Jackson Immuno Research Laboratories, Inc., West Grove, Pennsylvania, USA) or goat anti-mouse (111-035-144, Jackson Immuno Research Laboratories, Inc.) secondary antibodies and developed using enhanced chemiluminescence. The immunoblotted gCLCA1 protein was not detectable using antibodies gC1-N1 or gC1-C1 at any dilution used (S3 File).

### Immunocytochemistry of transfected HEK293 cells

Procedures are described in detail in S2 File. In brief, cells were grown on 8-well tissue chamber slides and transfected with *gCLCA1WT* or *EYFP-*mock plasmids. 72 h after transfection, the cells were briefly fixed in ice-cold methanol (100%) for five min followed by a fixation in 4% paraformaldehyde for ten min. The cells in each well were permeabilized with 0.1% Triton X-100 in PBS and blocked with 10% goat normal serum (GS) and 0.05% Tween 20 in PBS. After blocking, the cells were probed with untreated or pre-absorbed gC1-N1, or gC1-C1, or irrelevant affinity-purified rabbit polyclonal (anti-pig CFTR [81]) antibodies (each used at 1 μg/ml). Alexa fluor 568-conjugated goat anti-rabbit (AB_143157, Invitrogen, Carlsbad, California, USA) were used as secondary antibodies followed by 4′,6-diamidino-2-phenylindole (DAPI) nuclear counterstain for each experiment. No specific signals were detected using the gC1-N1 antibody (S3 File).

### Tissue expression pattern of gCLCA1 protein using immunofluorescence and immunohistochemistry

Methods are described in detail in S2 File. In brief, all tissues from the three animals that showed a *gCLCA1*-specific Ct-value below 35 were analyzed using immunofluorescence. FFPE-tissues were cut at 3 μm thickness, mounted on adhesive glass slides, and dewaxed. For immunohistochemistry, endogenous peroxidase was blocked by 0.5% H_2_O_2_. Antigens were retrieved using 1 mg/ml recombinant protease from *Streptomyces griseus* or microwave heating (600 W) for 15 min in 10 mM citric acid, pH 6.0, containing 0.05% Triton X-100. Slides were blocked with 10% Roti-ImmunoBlock and 20% GS in PBS and probed with gC1-C1 at 1 μg/ml or rabbit monoclonal anti-villin (ab130751, Abcam, Cambridge, United Kingdom) at 1:400 or irrelevant affinity-purified rabbit polyclonal (anti-pig CFTR [81]) at 1 μg/ml antibodies. 3,3´-Diaminobenzidine (DAB) stain was added after incubation with goat anti-rabbit biotinylated secondary antibodies (BA-1000, Vector Laboratories, Burlingame, California, USA) at 1:200 and an avidin-biotin complex solution. Slides were counterstained with 1% alcian blue in HCl at pH 1.0 and 0.1% nuclear fast red-aluminum sulfate.

For immunofluorescence analysis, tissue sections were permeabilized with 0.1% Triton X-100 in PBS and blocked with 10% GS and 0.05% Tween 20 in PBS. The slides were incubated with the gC1-C1 antibody at 1 μg/ml or an irrelevant affinity-purified rabbit polyclonal (anti-pig CFTR) antibody [81] using the same concentration. Alexa fluor 568-conjugated goat anti-rabbit (AB_143157, Invitrogen) secondary antibodies at 1:200 and a DAPI nuclear counterstain were used for immunofluorescence.

Unless otherwise noted, all *in vitro* studies were performed in a minimum of three independent cell batches where all transfections were conducted in parallel.

## Results

### The galline *gCLCA1* gene shares characteristics with its mammalian orthologues and encodes a putatively functional protein

**Fig 1:**
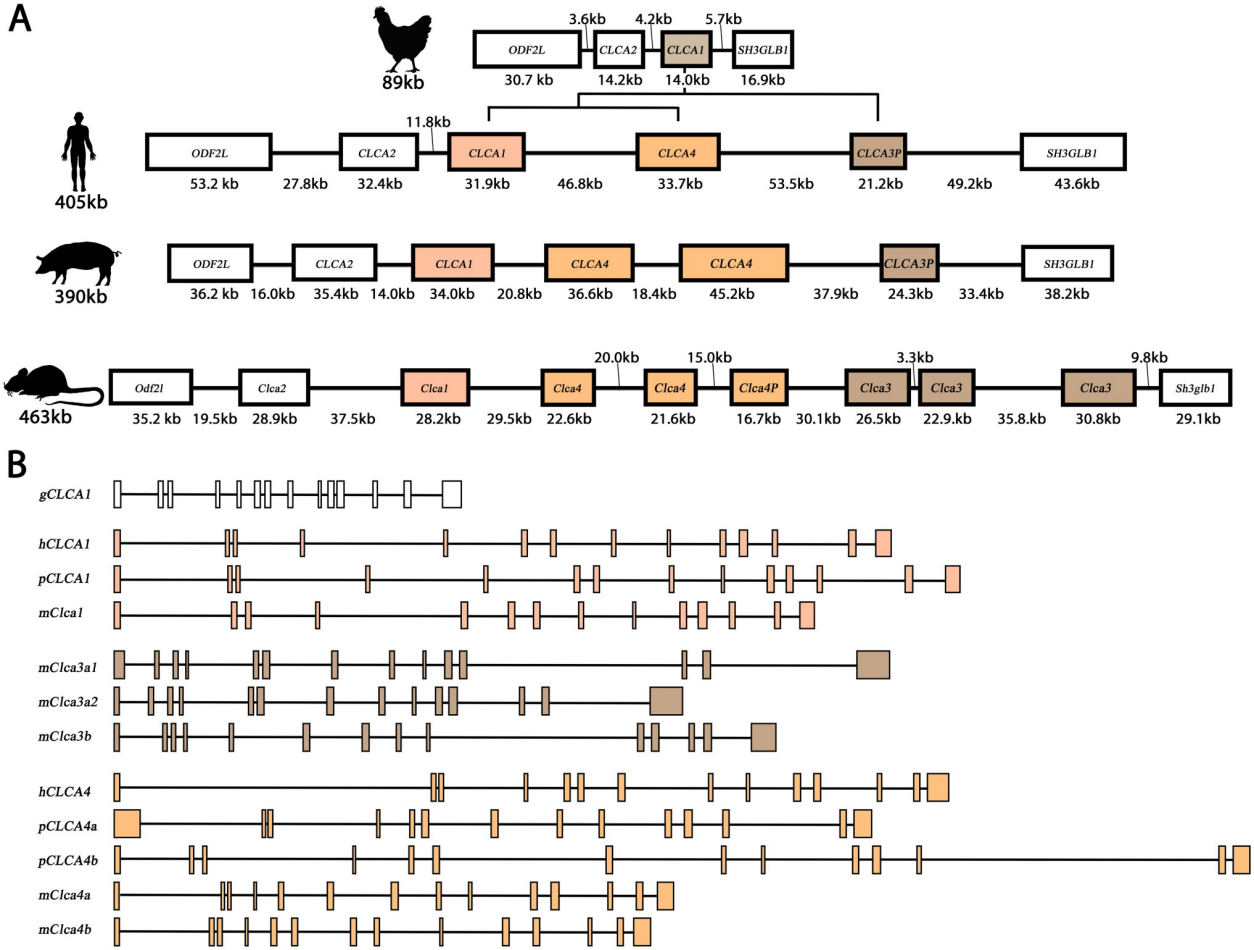
*gCLCA1* gene locus and structure as compared with mammalian homologues. (A) Comparisons of *CLCA* gene loci of the chicken and of the three mammalian representatives human, pig, and mouse. The galline *CLCA* locus consists of two *CLCA* genes, *gCLCA1* and *gCLCA2*, and is shorter than that of mammals. Genes are depicted as boxes and noncoding, intergenic segments are represented as black lines. The chicken locus is scaled 2-fold larger for illustrative purposes. P = pseudogene, kb = kilobases. (B) Comparative gene structure of *gCLCA1* and functional mammalian *CLCA1*, 3 and 4 genes. Like the mammalian *CLCA* genes of clusters 1, 3, and 4, *gCLCA1* is encoded by 14 exons (vertical boxes). Due to short intronic segments (horizontal black lines), the *gCLCA1* gene is shorter than its orthologues of the mammal cluster 1, 3, and 4.

The galline *CLCA* locus is located on chromosome 8 flanked by the genes *ODF2L* and *SH3GLB1* and it is shorter than mammalian *CLCA* loci ([49], Fig 1A). Compared to the human, porcine, and murine genes of cluster 1 ([49], Fig 1A and 1B), the *gCLCA1* gene is shorter, however, it is composed of 14 exons just as all intact mammalian *CLCA* genes (Fig 1B). The *gCLCA1* ORF consists of 2808 base pairs (bp, 936 aa), which is longer than those of mammalian homologues, such as human *CLCA1* (2742 bp, 914 aa) or murine *CLCA4b* (2775 bp, 925 aa) [64,82]. The predicted galline *CLCA1* gene does not contain premature stop codons or frameshift mutations as it had been identified for mammalian *CLCA3* [51] and therefore may encode a functional protein. Compared to the reference sequence of the NCBI Genbank (XM_422360.6, *Gallus gallus*), our *gCLCA1* ORF contained four synonymous and four non-synonymous single nucleotide polymorphisms (SNPs, S4 File).

### The gCLCA1 protein contains canonical CLCA domains

**Fig 2:**
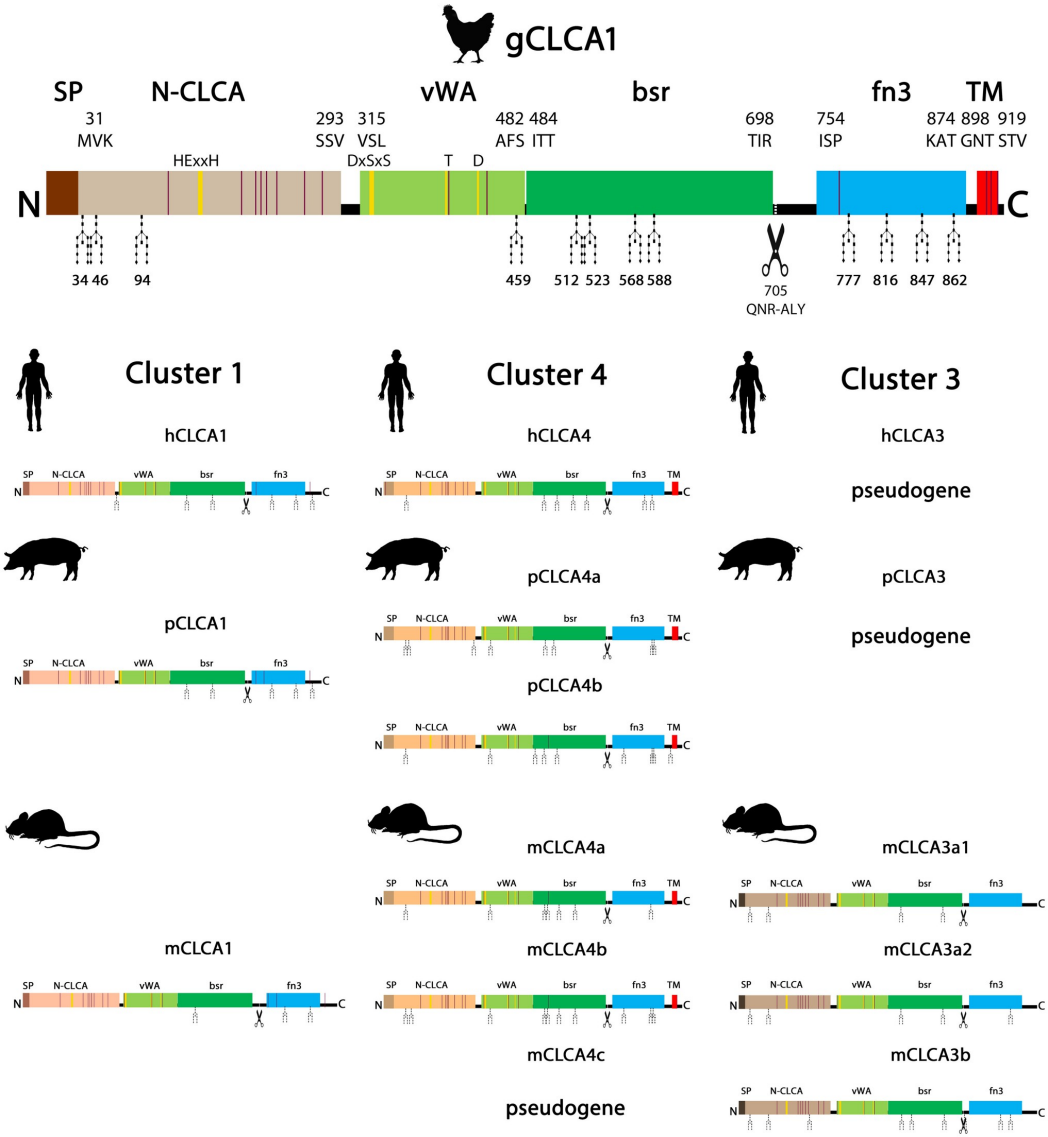
The basic functional domains of mammalian CLCA proteins are conserved in gCLCA1. Schematic depiction of the gCLCA1 protein and of CLCA proteins of clusters 1, 3, and 4 in three mammalian representatives (human, pig and mouse). A cleavable N-terminal signal peptide (dark brown box), an N-CLCA domain (N-CLCA, light brown box), a vWA-domain (vWA, light green box), a β-sheet rich domain (bsr, dark green box) and a fibronectin type III domain (fn3, light blue box) were predicted by *in silico* analyses and manual sequence alignments. The N-CLCA domain comprises an intact zinc-dependent metalloprotease motif (HExxH, vertical yellow bar). The vWA domain contains a MIDAS site (DxSxS, T, D, vertical yellow bar). Vertical dark red bars indicate cysteine residues, predominantly in the N-CLCA domain. The gCLCA1 protein is putatively cleaved after the amino acids QNR at aa position 705 (scissor). gCLCA1 has a putative C-terminal transmembrane domain (TM, light red box) similarly to the CLCA representatives of cluster 4. The gCLCA1 protein is an N-linked glycoprotein with 12 predicted glycosylation sites. The number of glycosylation sites is higher than in the CLCA proteins of cluster 4, followed by the proteins of cluster 3 and 1. Putative glycosylation sites are indicated by 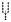-icons. Numbers display the absolute position, for protein domains further specified by the first or last three amino acids of each domain, respectively.

All known mammalian CLCA proteins enter a secretory pathway and contain an N-CLCA (PFAM identifier: pfam08434) and a vWA (PFAM identifier: pfam13519) domain. For gCLCA1, the SignalP 5.0 algorithm also predicted a signal peptide between aa1 and 29. Furthermore, the NCBI Conserved Domain Database (CDD), EMBL-EBI HMMER and SUPERFAMILY 2 databases identified an N-CLCA (aa31 to 293) and a vWA domain (aa315 to 482, Fig 2). A cysteine-rich domain within the N-terminal cleavage product, which has been described for mammalian CLCA proteins [77], was also identified in gCLCA1 (Fig 2).

**Fig 3:**
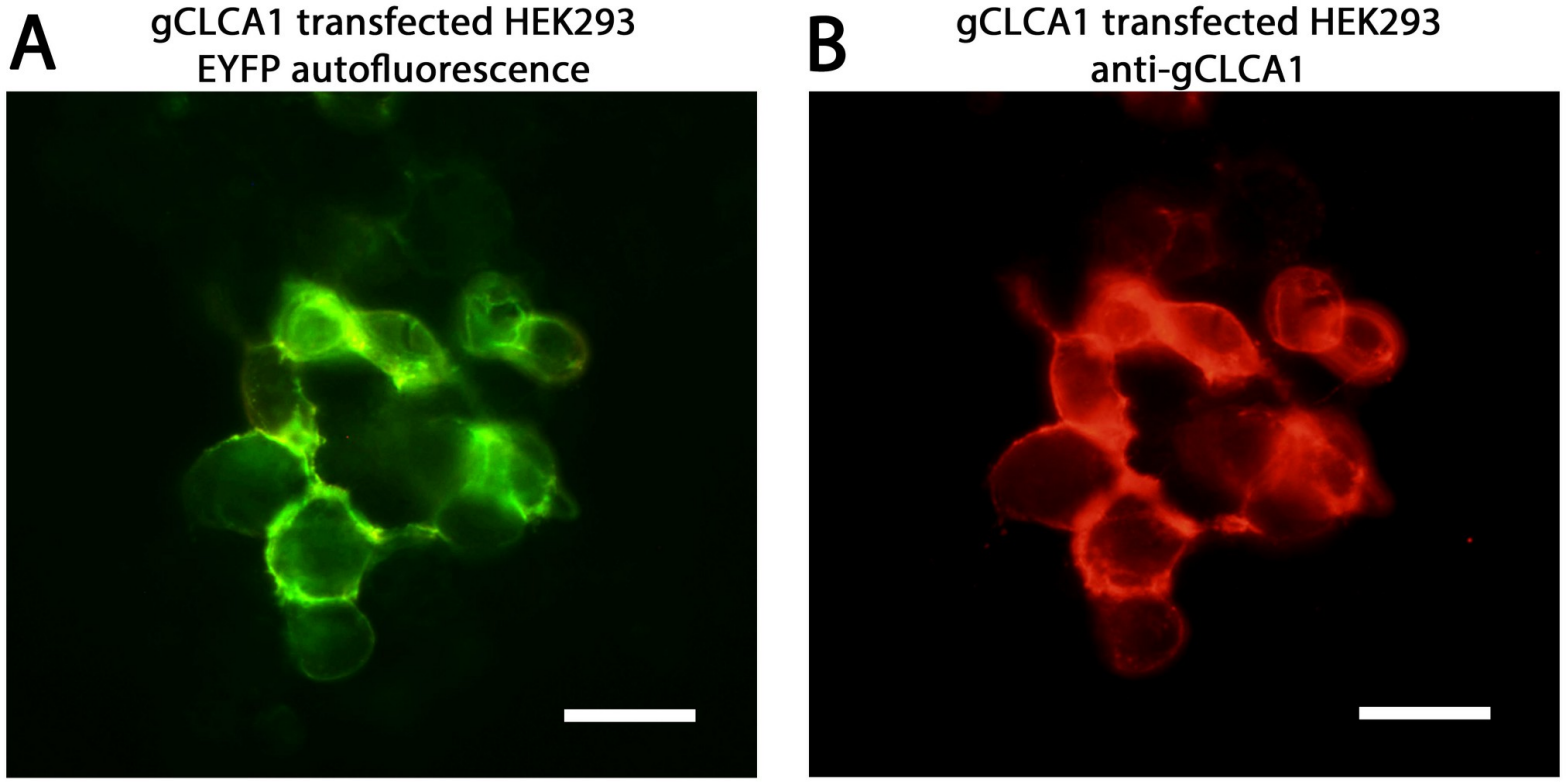
Localization of the C-terminal cleavage product of gCLCA1 in the plasma membrane. (A) The C-terminal cleavage product of the gCLCA1EYFP fusion protein was detected at the plasma membrane of transiently transfected HEK293 cells via autofluorescence of the EYFP-tag (green). (B) A corresponding red signal was reproducible by immunofluorescence using the gC1-C1 (anti-gCLCA1 C-terminal) antibody. Alexa fluor 568-conjugated secondary antibodies. Bars indicate 20 μm. Representative images of three independent experiments.

Similar to the transmembrane proteins of the mammalian cluster 4, the *in silico* analysis consistently predicted a single hydrophobic transmembrane domain between aa897 (SOSUI, Phobius) / 898 (CCTOP) and aa919 (SOSUI) / 922 (CCTOP, Phobius) (Fig 2) for gCLCA1. To verify the presence of the predicted transmembrane domain, the cellular location of the C-terminal cleavage product of the gCLCA1 protein was experimentally analyzed. A green signal of the C-terminal EYFP-tag was detected along the plasma membrane of cells transfected with the *gCLCA1WT* plasmid (Fig 3A). This is in contrast to the diffuse green, cytoplasmic autofluorescent EYFP signal as detected in cells transfected with the EYFP-mock plasmid (S6 File). pcDNA3.1+ transfected HEK293 cells failed to show a specific green autofluorescent signal (S6 File). A red signal along the plasma membrane was found by immunostaining of the cells with the gC1-C1 antibody which binds to the C-terminal subunit of gCLCA1 (Fig 3B, S3 File). These results suggest the presence of a C-terminal transmembrane domain in gCLCA1 similar to mammalian CLCA4 proteins.

### The gCLCA1 protein has biochemical properties similar to those of mammalian CLCAs Posttranslational Cleavage

**Fig 4:**
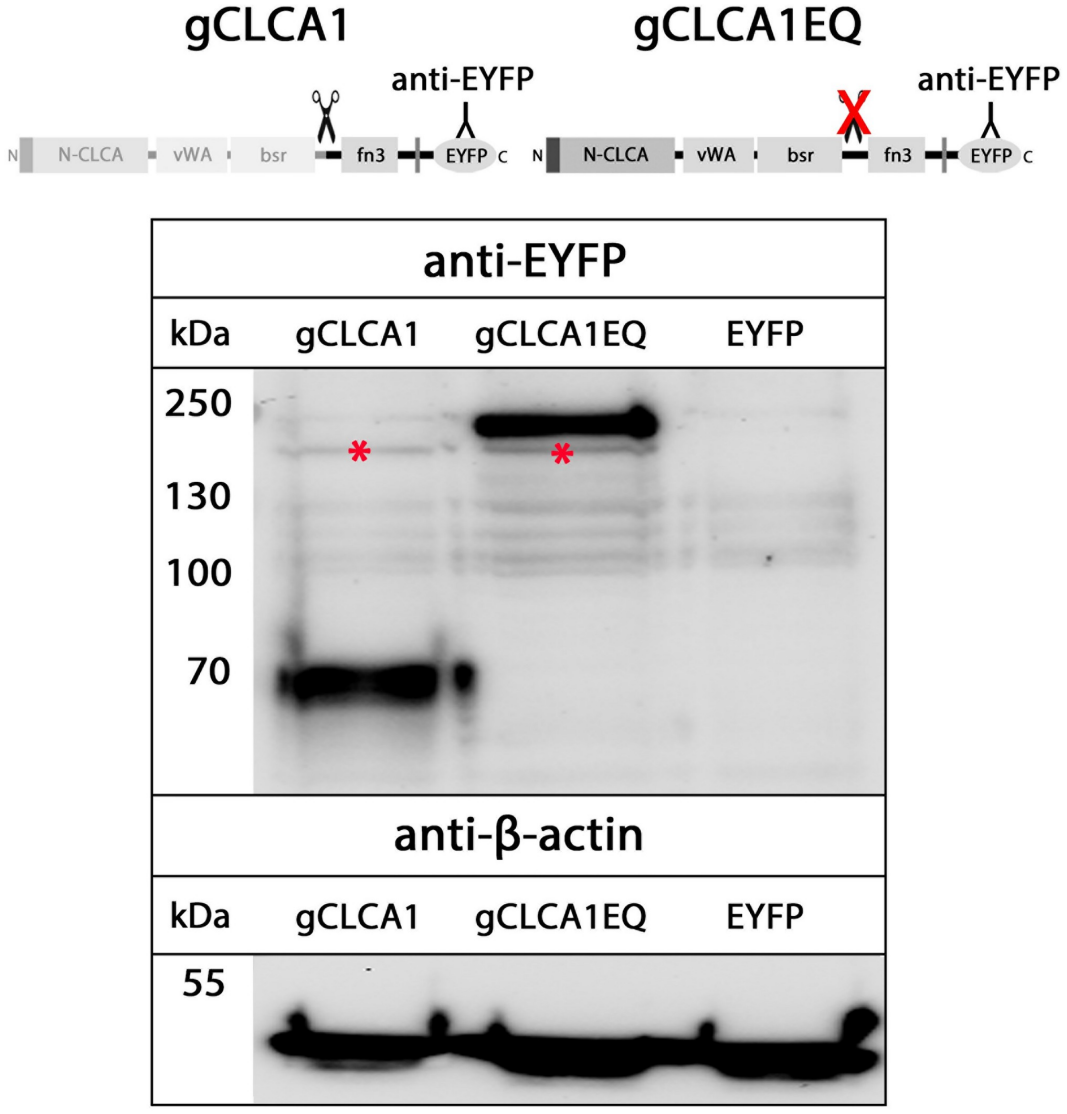
gCLCA1 is a HExxH-dependent cleavable protein. Immunoblot of cell lysates from HEK293 cells transiently transfected with the *EYFP*-mock plasmid (EYFP), the *gCLCA1WT* (gCLCA1) plasmid, and the *gCLCA1E164Q* plasmid containing an EQ substitution at position two of the catalytic active HExxH motif (gCLCA1EQ) is shown. A C-terminal cleavage product of gCLCA1 and its immature glycosylated precursor protein were identified at ~64 kDa or at ~154 kDa (*), respectively. Cleavage was prevented by the EQ substitution in the HExxH motif as no cleavage product was detectable; however, a strong band at ~166 kDa was identified, which putatively represents the uncleaved, mature glycosylated full-length protein. Identical to cells transfected with the *gCLCA1WT* plasmid, the immature glycosylated precursor protein at ~154 kDa was detected in cell lysate from *gCLCA1E164Q* transfected cells. To control for equal total protein loading the samples were identically immunoblotted with primary anti-beta-actin antibodies. Representative images of three independent experiments.

**Fig 5:**
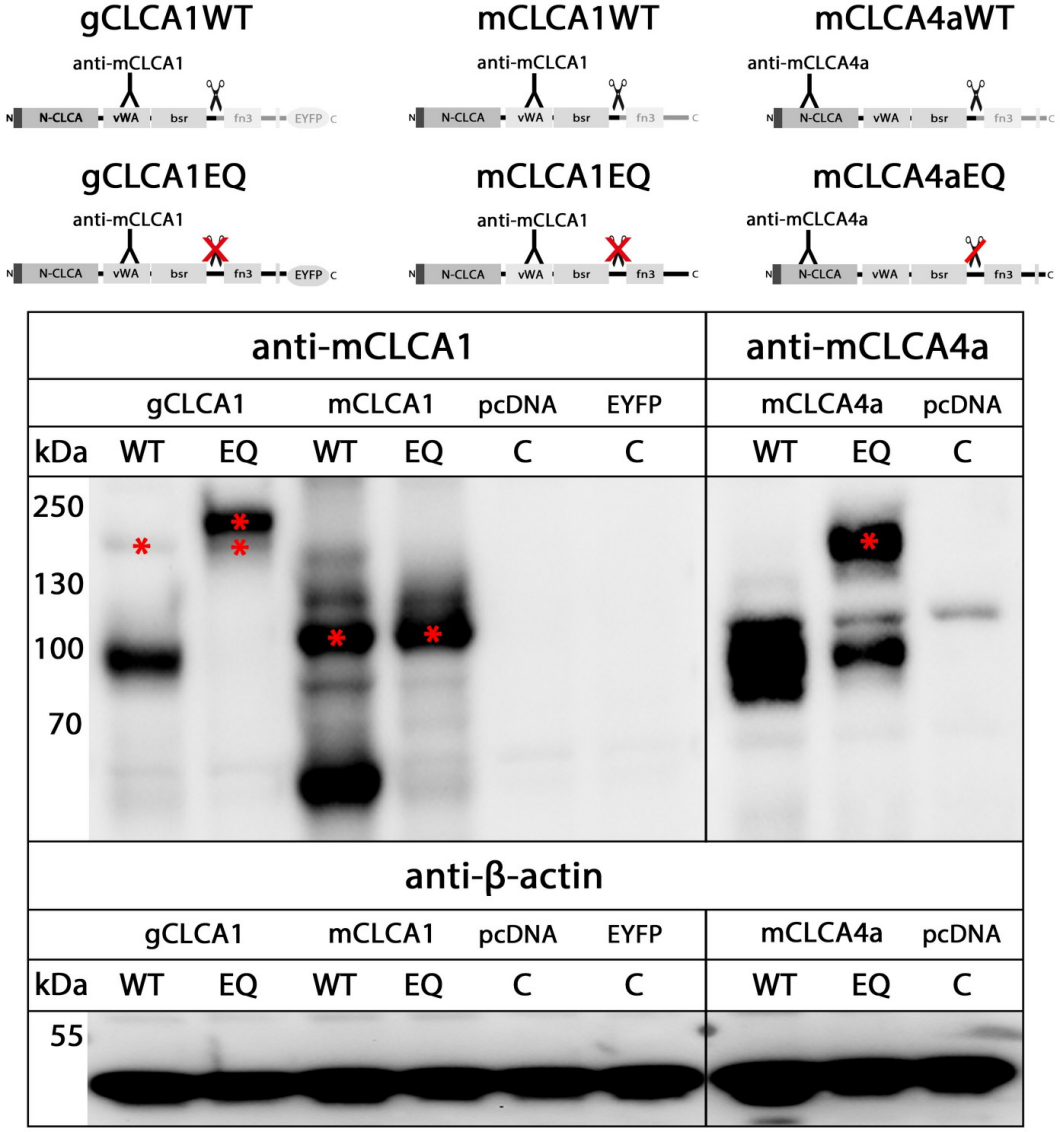
The gCLCA1EQ mutant eliminated autocatalytic cleavage similar to CLCA proteins of mammalian cluster 1. Immunoblotting of HEK293 cell lysates. The left panel illustrates immunoblots from murinized galline CLCA1, the central panel from murine CLCA1 and the right panel from murine CLCA4a constructs. The proteins of the three homologues differ in their molecular weights. The mutation of the HExxH motif of gCLCA1 eliminated the cleavage of the ~154 kDa precursor protein (*) similarly to the murine CLCA1 protein. In contrast, the cleavage of the murine CLCA4aEQ mutant was only impaired, but not totally absent. Asterisks (*) indicate the uncleaved protein of the respective CLCA homolog. To control for equal total protein loading, the samples were identically immunoblotted with primary anti-beta-actin antibodies. WT = HExxH wild type motif, EQ = EQ mutation in the HExxH motif, C = mock-transfected control. Representative images of three independent experiments.

The zinc-binding aa motif HExxH of the CLCA domain, which is necessary for autocatalytic cleavage of a precursor protein into a larger N- and a smaller C-terminal cleavage product [64], is a common feature of all known mammalian CLCA proteins [77,83]. An intact HExxH motif (HEWAH, aa163–167) and a putative proteolytic cleavage site (QNR/ALY, after R705) were also identified in the gCLCA1 protein (Fig 2). It has been reported that mutagenic disruption of the mammalian HExxH motif abrogates the cleavage of mammalian CLCA1 or only impairs that of CLCA4 proteins [78,80]. To evaluate a putative HExxH-dependent cleavage of gCLCA1, lysates from cells transfected with the gCLCA1 wild type and the gCLCA1E164Q mutant were immunoblotted with anti-EYFP antibodies. For cells expressing the wild-type protein, a weak band of the precursor protein at ~154 kilodalton (kDa) and a strong band of the C-terminal cleavage product at ~64 kDa were detected (Fig 4). In contrast, no cleavage product was visible in lysates from cells transfected with the gCLCA1E164Q mutant. A single strong band of the uncleaved, mature glycosylated protein at ~166 kDa and the weak band of the immature glycosylated precursor protein at ~154 kDa (Fig 4) were detected. These findings are consistent with an autocatalytic cleavage of gCLCA1 as previously described for mammalian CLCAs. Additionally, the EQ mutation of gCLCA1 leads to a total abrogation of the cleavage event similar to mammalian CLCA1 (Fig 5).

### Secretion of the N-terminal cleavage product

**Fig 6:**
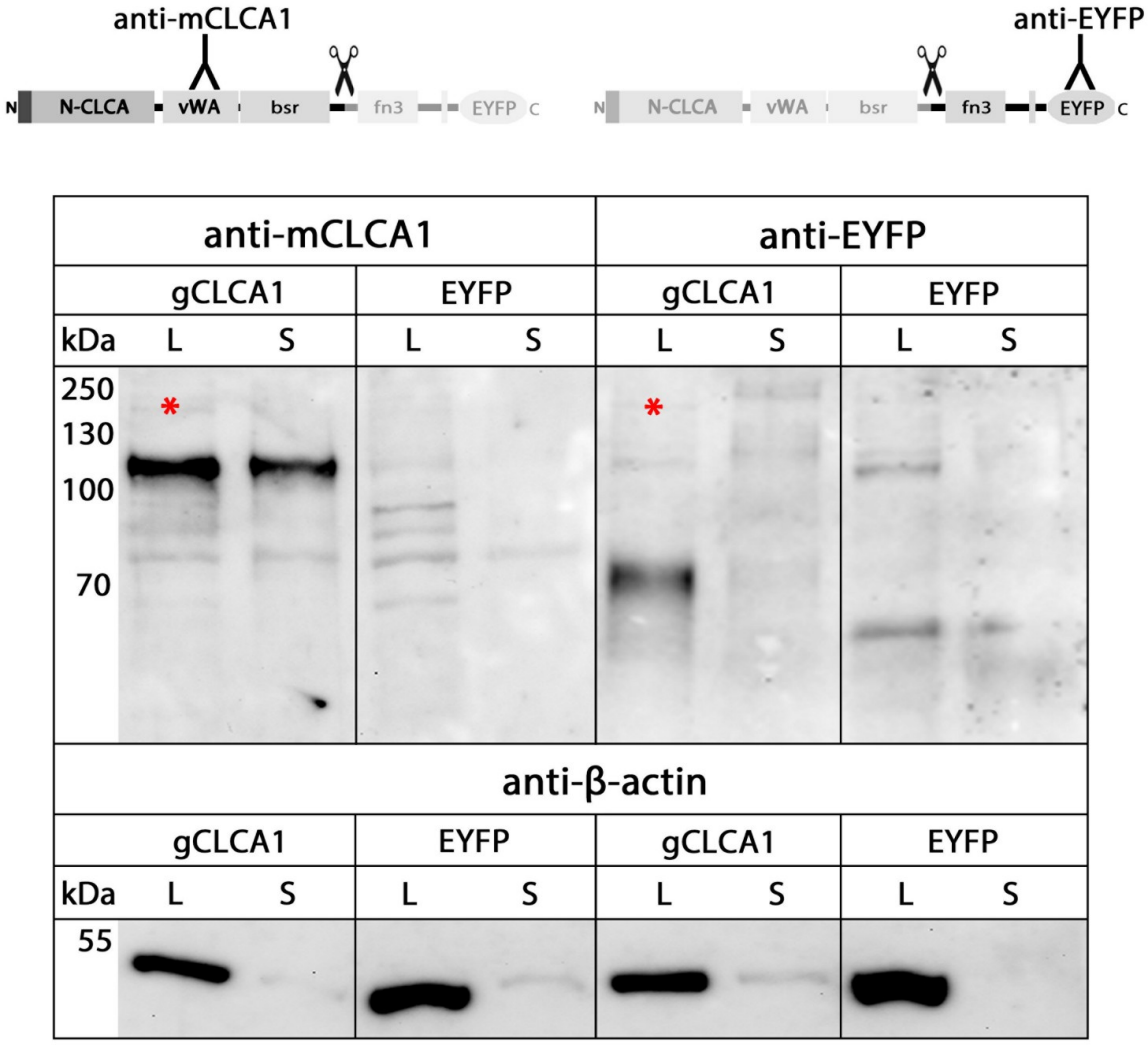
Secretion of only the N-terminal cleavage product of gCLCA1 into the cell culture supernatant. Immunoblotting of lysates (L) and cell culture supernatants (S) from HEK293 cells transfected with the *gCLCA1Nmabc1* (gCLCA1) and *EYFP*-mock plasmid (EYFP). The N-terminal cleavage product was detected at ~102 kDa in cell lysate and supernatant. In contrast, the C-terminal, transmembrane domain containing cleavage product was exclusively detected at ~64 kDa in the cell lysate, but not in the supernatant. A weak band of the immature glycosylated gCLCA1 precursor protein was detectable in the cell lysate at ~154 kDa (*). The same amounts of proteins for the respective lysates and supernatants from transfected cells were analyzed in these experiments. To control for equal total protein loading of cell lysates and for lack of contamination of the supernatants by cell debris, the samples were identically immunoblotted with primary anti-beta-actin antibodies. Representative images of three independent experiments.

It has been shown that the N-terminal subunit of mammalian CLCA4 is secreted [46,62]. To test for secretion of the N-terminal cleavage product of the gCLCA1, conditioned cell culture media and lysates from cells transfected with *gCLCA1* were analyzed. The ~102 kDa N-terminal cleavage product was detected in the supernatant and the cell lysate while the ~64 kDa C-terminal cleavage product was exclusively found in the cell lysate (Fig 6), which further supports the presence of a C-terminal transmembrane domain. Consistently, only tryptic peptides from the N-terminal cleavage product were found in the supernatant from cells transfected with the gCLCA1 using LC-ESI-MS/MS (S5 File). Thus, similar to mammalian CLCA4 proteins, the N-terminal cleavage product of gCLCA1 was secreted.

### N-glycosylation and cleavage in the medial Golgi

**Fig 7:**
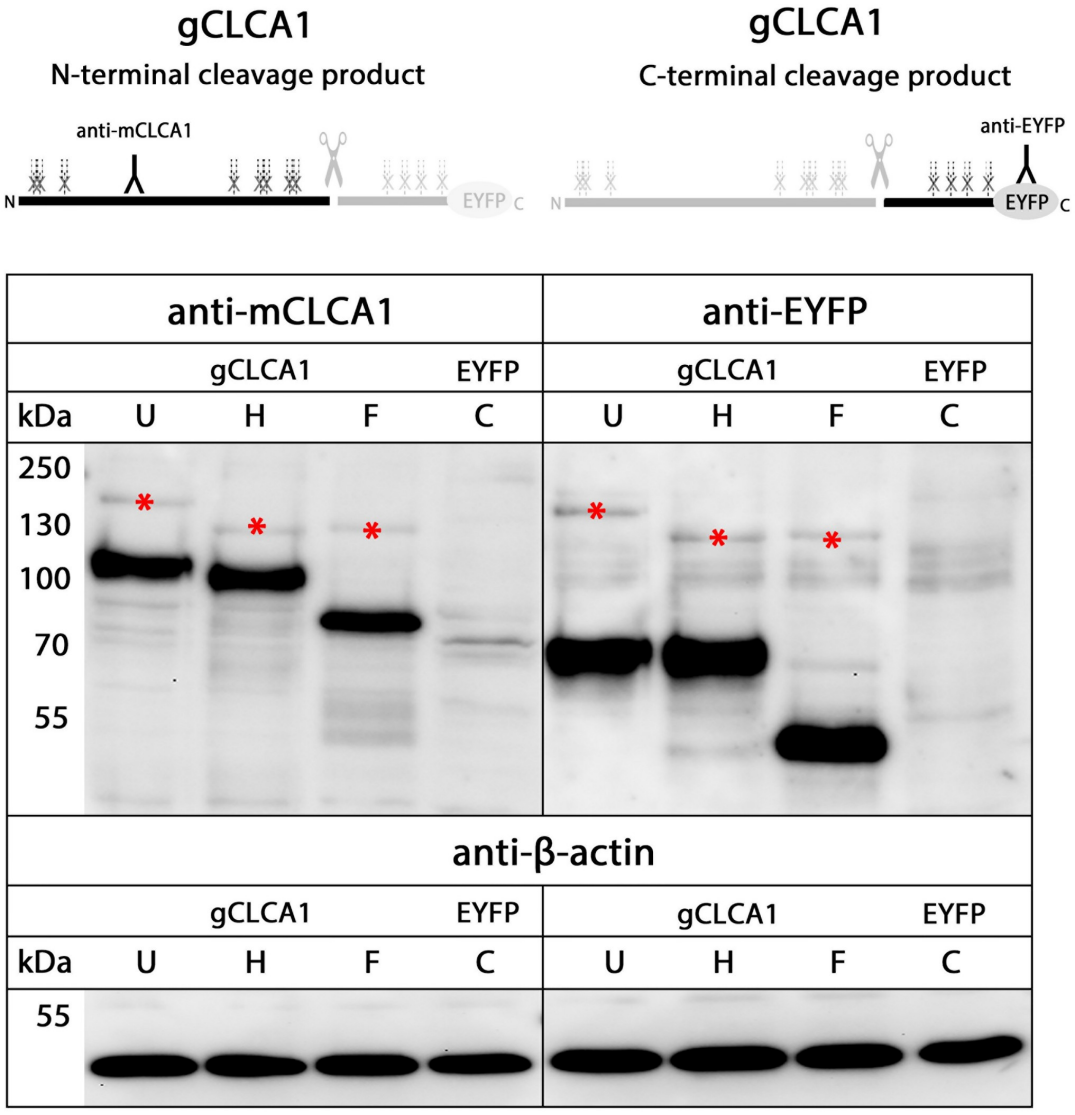
Glycosylated gCLCA1 is cleaved in the medial Golgi. Immunoblot of cell lysate of HEK293 cells transiently transfected with the *gCLCA1Nmabc1* plasmid (gCLCA1) and a control *EYFP*-mock plasmid (EYFP). The gCLCA1 precursor (*) was mannose-rich glycosylated, reduced in size by endo H and PNGase F treatment. In contrast, both cleavage products were solely sensitive to PNGase F as shown by a reduction in size of the N-terminal and C-terminal cleavage product to ~78 kDa or ~52 kDa, respectively. The cleavage event seems to occur in the medial Golgi. U = Untreated, C = untreated mock-control, H = endo H treated, F = PNGase F treated cell lysate. To control for equal total protein loading, the samples were identically immunoblotted with primary anti-beta-actin antibodies. Representative images of three independent experiments.

Mammalian CLCA proteins of clusters 1, 3, and 4 are N-linked glycoproteins, with different number of glycosylation sites [46,49,51,52,62,66]. Consistent with these results, the NetNGlyc 1.0 algorithms predicted 12 potential N-linked glycosylation sites for gCLCA1, located at aa positions 34, 46, 94, 459, 512, 523, 568, 588, 777, 816, 847, and 862 (Fig 2). To corroborate these predictions, cell lysate from *gCLCA1Nmabc1-*transfected cells was treated with endoglycosidases endo H and PNGase F for identification of the kind and extent of the glycosylation. The ~154 kDa precursor protein was sensitive to endo H and PNGase F (Fig 7) resulting in a size shift from ~154 kDa to ~130 kDa which shows an immature high mannose-type glycosylation pattern. In contrast, the N- and C-terminal cleavage products were resistant to endo H but sensitive to PNGase F treatment, shown by a reduction in size from ~102 kDa to ~78 kDa (Fig 7) and ~64 kDa to ~52 kDa (Fig 7), respectively. This indicates that the majority of the predicted eight consensus glycosylation sites in the N-terminal subunit and four glycosylation sites in the C-terminal cleavage product may be used for glycosylation, when a molecular weight of ∼3 kDa per site is assumed [84]. Furthermore, the complex, high mannose-rich glycosylation pattern of the N- and C-terminal cleavage products, in contrast to the precursor protein, suggests a cleavage of gCLCA1 in the medial Golgi.

### The gCLCA1 protein is expressed in enterocytes throughout the intestine

**Fig 8:**
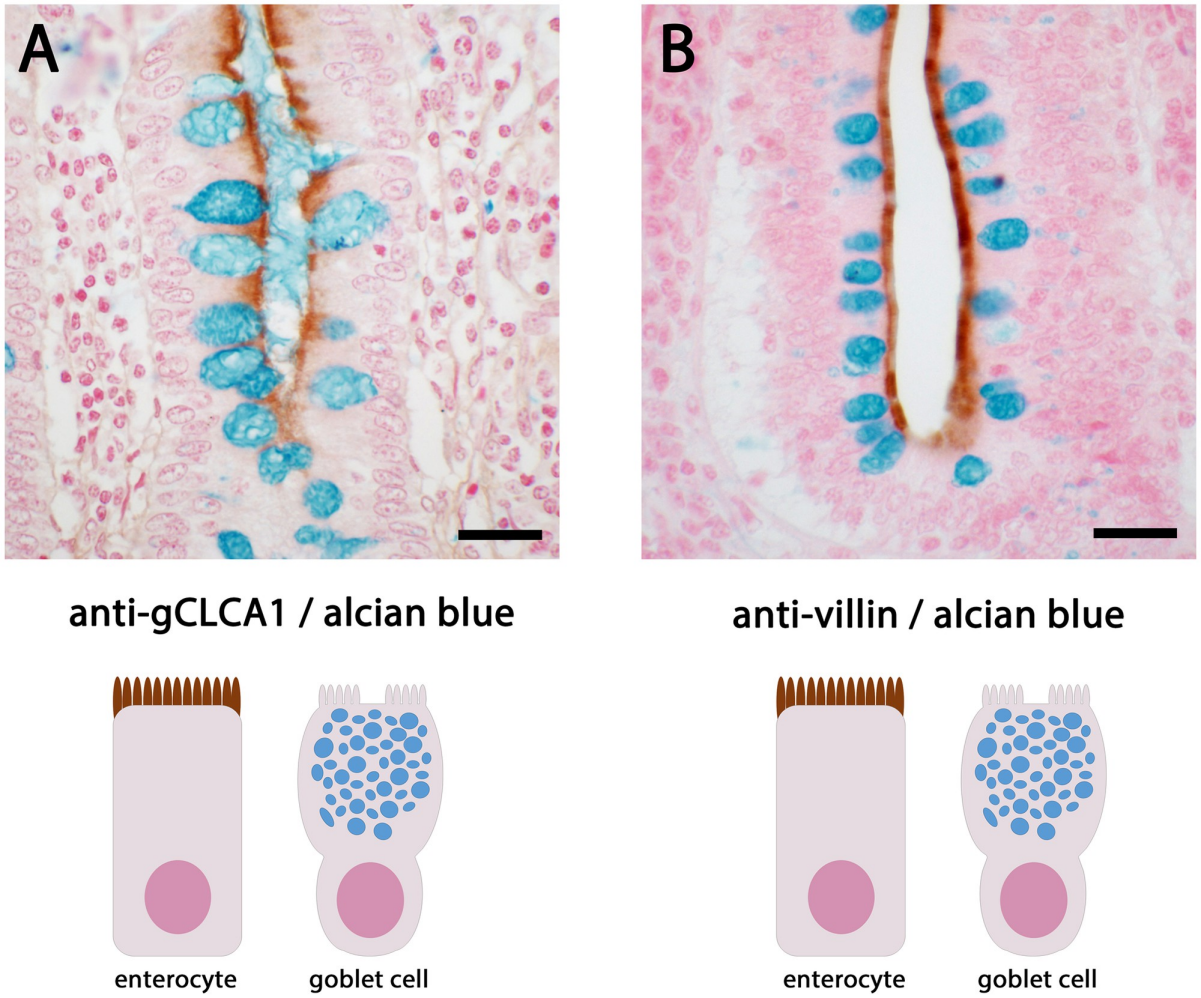
gCLCA1 protein is expressed in the brush border membrane of enterocytes, but not in goblet cells. (A) The gCLCA1 protein (brown) was detected at the apical surface of enterocytes of colonic crypts by immunohistochemistry using the gC1-C1 (anti-gCLCA1 C-terminal) antibody. A counterstain with alcian blue highlights goblet cells (dark blue) that did not show any gCLCA1 protein expression. (B) The cellular expression pattern was similar to villin, a marker for the brush border of enterocytes (brown). Color was developed using DAB as substrate (brown). Bars indicate 20 μm.

**Fig 9:**
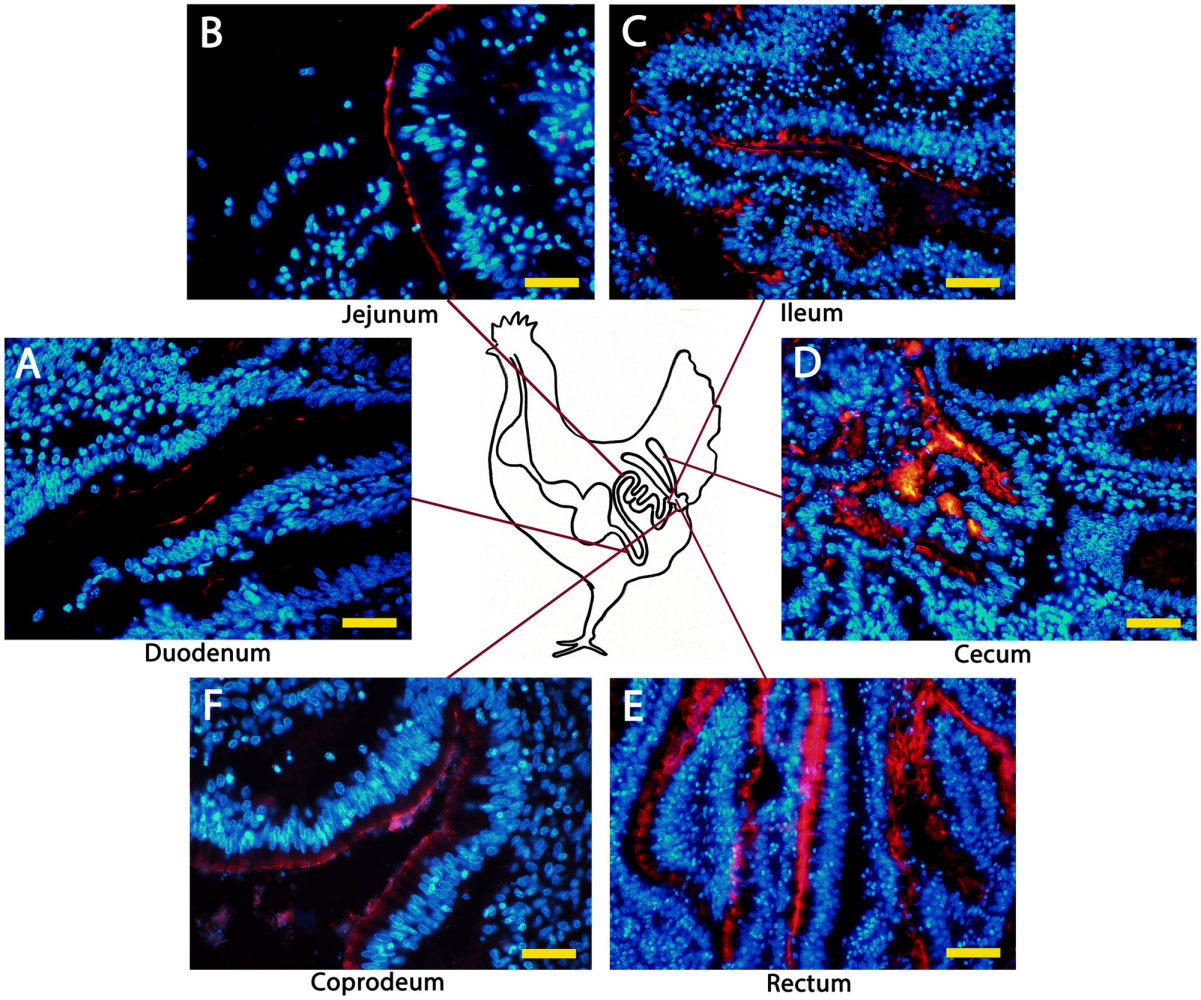
gCLCA1 protein is expressed along the chicken intestine. Anti-gCLCA1 C-terminal antibody was used to detect gCLCA1 along the epithelium in duodenum (A), jejunum (B), ileum (C), cecum (D), rectum (E) and coprodeum (F). Alexa fluor 568-conjugated secondary antibodies and DAPI counterstain (blue). Bars indicate 40 / 100 μm.

The expression pattern of gCLCA1 was identified on the mRNA and protein levels. *gCLCA1* mRNA was detected in all segments of the alimentary tract (pharynx, esophagus, duodenum, jejunum, ileum, cecum, rectum, coprodeum), the Bursa of Fabricius, the eye, and the lung (S1 File). These tissues were further analyzed via immunofluorescence and immunohistochemistry to identify the cell types that express gCLCA1. The protein was exclusively found at the brush border of intestinal epithelial cells (Fig 8A), which also express villin, a structural marker of enterocytes (Fig 8B, [85]). However, mucin-producing intestinal goblet cells lacked any gCLCA1 expression (Fig 8A). In all segments of the intestinal tract, gCLCA1 protein was detected in enterocytes (Fig 9). Additionally, it was detected along the bursal epithelium (S7 File). No specific gCLCA1 protein signals were detected in any other tissue analyzed. Compared to the expression pattern of CLCA proteins in mammals, the expression of *gCLCA1* showed congruence with mammalian CLCA4 proteins [46,48,62].

### The phylogenetic signal of full-length gCLCA1 scatters on the domain level

**Fig 10:**
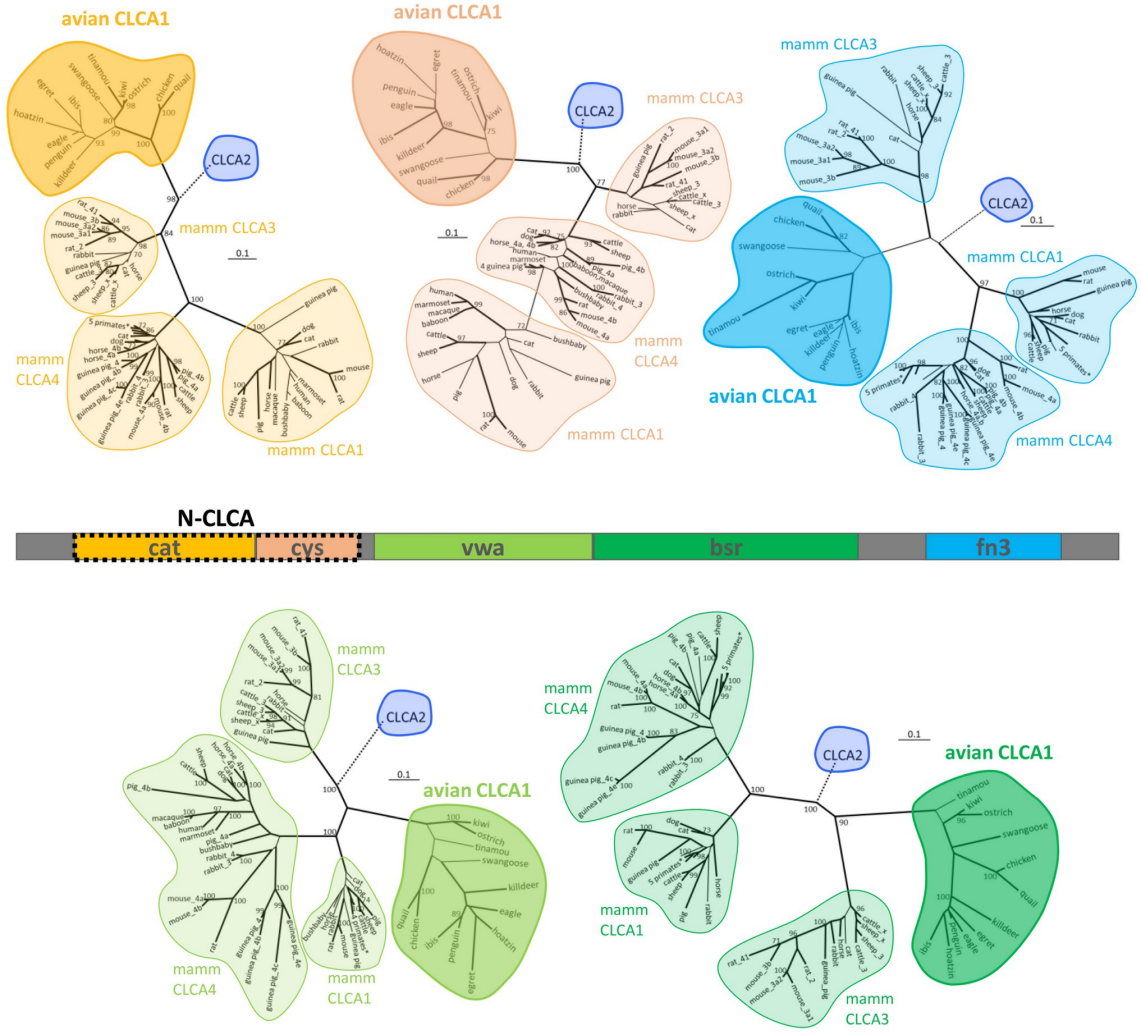
Phylogenetic relationship of CLCA proteins. Maximum likelihood (ml), most parsimony (mp) and 100 neighbor joining (nj) trees were generated for the five distinct CLCA domains and merged. Tree was based on the ml tree, with branches in thick, when occurring in the mp tree and the branch nodes indicated when occurring in more than 70 nj trees. CLCA2 orthologues were reduced to an outgroup. Other clusters were highlighted. To avoid confusion due to limited space, the five primate species analyzed (human, macaque, baboon, marmoset, bushbaby) are occasionally not separately defined (*). In the case of CLCA duplicates in mammalian species, the sequences are designated by the abbreviation given in GenBank (i.e. “mouse_3a2” for murine Clca3a2) or by “x” if the available sequence has been designated as “CLCA-like” or “unknown”.

Phylogenetic analyses of five protein domains reveal a clustering of the CLCA proteins in these domains (Fig 10). Designated mammalian and avian CLCA2 form a outgroup from the avian CLCA1 and mammalian CLCA1, 3, 4. Any of the sequences cluster with the same group throughout the protein. The hierarchy within the mammalian clusters is not fully consistent, due to the known evolutionary dynamics of the rapid formation of major mammalian branches such as primates, rodents, ungulates, or carnivores within a short period of time and the independent expansion at different paces later on. Similar processes seem to have taken place in avian diversification [86] and accordingly, the relationship between the analyzed avian species remains elusive. Only close relationships such as between chicken and quail and, with some restrictions, kiwi, ostrich and tinamou, are consistently reflected throughout the CLCA protein. Adding to the separation of the analyzed clusters from the CLCA2 family, mammalian CLCA1 and CLCA4 form a major branch, independent from avian CLCA1 and mammalian CLCA3. The analysis of the five domains shows variable genetic distances among avian CLCA1 and mammalian CLCA3 and CLCA1/4. The relationship between CLCA2, avian CLCA1, mammalian CLCA3 and CLCA1/4 is therefore inconsistent throughout the full-length protein.

## Discussion

In contrast to the mammalian *CLCA* gene family, which consists of up to eight members, there are only two *CLCA* homologues in chicken [51], suggesting that the mammalian and the avian CLCA loci have been subjected to different evolutionary pressures. *CLCA2* is conserved in mammalian and chicken loci [51], whereas the galline CLCA1 displays low genetic distance to all other three mammalian CLCA proteins. Here, we characterized gCLCA1 as a putative prototype of the avian class in terms of protein structure, biochemical properties, and expression pattern and compared the results with the nature of the mammalian CLCA 1, 3 and 4 proteins. The gCLCA1 protein shares features of all CLCA family members such as a signal peptide, a CLCA domain with a HExxH motif, a vWA domain, and an autocatalytic cleavage site (Fig 2, Table 1). The phylogenetic analysis revealed separated CLCA clusters, an evident separation of the avian and mammalian CLCA2 from the other clusters and a consistent relationship between the mammalian CLCA1 and CLCA4 clusters. The position of avian CLCA1 in the phylogenetic tree was difficult to interpret, confirming the previously postulated idea of high dynamics of mammalian CLCA clusters 1, 3, and 4 [51]. It appears that avian CLCA1 and its mammalian relatives may have developed independently from a common ancestor, the avian CLCA1 thus sharing select properties of mammalian CLCA 1, 3, and 4.

**Table 1:** Comparison of gCLCA1 with the mammalian CLCA 1, 3, and 4.

*Trait*	*gCLCA1*	*Mammalian CLCA 1*	*Mammalian CLCA 3*	*Mammalian CLCA 4*
*Number of exons*	*14*	*14*	*14*	*14*
*Duplication of genes*	-	-	+	+
*Pseudogenization*	-	-	+	(+)
*Signal peptide*	+	+	+	+
*N-CLCA domain*	+	+	+	+
*HExxH motif*	+	+	+	+
*vWA domain*	+	+	+	+
*Canonical cleavage site*	+	+	+	+
*TM domain*	+	-	-	+
*N-terminal subunit secreted in vitro*	+	+	+	+
*C-terminal subunit secreted in vitro*	-	+	+	-
*Posttranslational cleavage*	+	+	+	+
*EQ mutation abrogates cleavage*	+	+	*n*.*t*.	*- (* * *)*
*N-glycosylation sites*	+++	+	++	+++
*Protein expressing cell type*	*Enterocyte*	*Mucin-producing cells of intestinal*, *airway and female reproductive tract*	*Ciliated epithelial airway cell* *mucus producing airway submucosal cell* *keratinocyte* *endothelial cell* *smooth muscle cell*	*Enterocyte* *ciliated epithelial airway cell*

* only partially inhibited.

n.t.: Not tested.

N: Amino terminal.

C: Carboxy terminal.

vWA: Von Willebrand factor type A.

TM: Transmembrane.

Regarding the cellular expression pattern, gCLCA1 shares more properties with the CLCA4 proteins than with CLCA proteins 3 and 1 (Table 1). gCLCA1 protein was detected at the apical surface of enterocytes along the alimentary canal, and this expression pattern mirrors that of mammalian CLCA4 proteins [46,48,62]. In contrast, mucin-producing goblet cells, which abundantly express CLCA1 in all mammalian species investigated [2,3,49,50,53], did not appear to express gCLCA1. Recently, it has been shown that murine CLCA1 has mucus-processing properties [13] and controls mucus expansion in the colon [12]; the presence of a predicted equivalent protease motif in the secreted N-terminal cleavage product of gCLCA1 suggests that this protein may participate in mucus homeostasis in the chicken intestine. In addition, we found gCLCA1 protein in the epithelial lining of the cloaca and the Bursa of Fabricius, the latter is an immunological organ unique to birds. *gCLCA1* mRNA was present in other organs, such as pharynx, esophagus, eye, and lung, but this was not accompanied by detectable gCLCA1 protein levels.

The presence of a transmembrane domain in the C-terminal subunit, and the secretion of only the N-terminal subunit are other traits shared by gCLCA1 and mammalian CLCA4 proteins. In contrast, mammalian CLCA1 and CLCA3 are soluble proteins and secreted in their entirety [3,64–66]. The function of the different gCLCA1 protein domains as well as the relevance of its glycosylation remain to be investigated.

gCLCA1 constructs with a modified HExxH motif lacked posttranslational autocatalytic cleavage abilities, as reported for mutated human, murine and porcine CLCA1 [77,78]; in contrast, equivalent mutations in the HExxH domain of CLCA4 impair autocatalysis only partially [80]. Thus, whereas gCLCA1 appears to share tissue distribution and cellular expression patterns with mammalian CLCA4, regarding autocatalytic properties it would be functionally closer to mammalian CLCA proteins of cluster 1.

### Which characteristics might a common ancestor of gCLCA1 and the mammalian CLCA 1, 3 and 4 proteins have had?

The protein and expressional characteristics of gCLCA1 and the comparison with its mammalian relatives allow us to speculate about the nature of their evolutionary ancestor. The commonly identified traits of gCLCA1 and the mammalian CLCA4 proteins, such as “proteolytic cleavage”, “anchoring of the C-terminal subunit in the plasma membrane via a transmembrane (TM) domain”, “secretion of the N-terminal subunit”, glycosylation and enterocyte-exclusive expression (Table 1) might be considered as a molecular symplesiomorphy and thus be shared also by a common ancestor.

Such a hypothesis might gain some support from more distantly related species. *In silico* analysis of the 940 aa *chloride channel accessory 4 gene 1* (CLCA4.1, NP_001267595.1), a CLCA homologue from *Xenopus laevis*, suggested heavy glycosylation and a strongly hydrophobic C-terminus consistent with a transmembrane domain. RT-qPCR analysis had identified the gut as an expressing tissue, however, the expressing cell type of this CLCA member is still unknown [87]. Our comparison of the aa sequence of Xenopus CLCA4.1 (xCLCA4.1) with gCLCA1 and proteins of the mammalian CLCA 1, 3, and 4 identified a proteolytically active HExxH site as well as a canonical cleavage site at position 696 (RSR-ALY) in the amphibian CLCA member, which suggests a putative posttranslational cleavage of the protein. Thus, a common CLCA ancestor might have been a glycosylated and cleavable transmembrane protein expressed in the intestine.

The three mammalian *CLCA* 1, 3, and 4, seem to have developed from duplication events that did not happen in chicken. Gene duplication, as a key driver of evolution [88], is documented in nine vertebrate species for at least 37,000 genes [89]. Either, as the most common case [90], the duplication is completely lost or the duplicated gene becomes inactivated, resulting in pseudogenization. Pseudogenization is known for the *CLCA3* gene in certain mammals such as dry-nose primates, pigs, and sheep [51]. On the other hand, the duplication can be maintained and adapted, resulting in the gain of a new function (neofunctionalization) or the original function is now shared among both, the original and the duplicated gene (subfunctionalization) [90]. Additional species-specific duplication events of clusters 3 and 4 have been described in mammals. Three and two intact *CLCA3* genes are present in the murine or bovine CLCA locus, respectively [51]. Duplication of the *CLCA4* gene has also been identified in the pig and the mouse [51]. The duplication of functional *CLCA* genes in mammals seems to amplify the expression pattern of this gene family. In contrast to the expression of the galline CLCA1 and mammalian CLCA4 proteins in enterocytes, CLCA1 proteins are expressed by mucin-producing cells such as goblet cells of the intestinal and respiratory tract [2,3,49,53]. The expression pattern of the duplicated CLCA3 members is broader than of other clusters [51] and is not restricted to epithelial cells. CLCA3 members are also expressed in cells derived from the mesoderm such as endothelial or smooth muscle cells [42]. In addition to the diversity of the cellular expression pattern of mammalian CLCA proteins, structural variations in terms of the lack of a transmembrane domain are evident in the CLCA proteins 1 and 3 compared to 4 and the gCLCA1. The structural differences of the CLCA proteins and the diverse expression pattern may reflect that the *CLCA* gene duplication events in mammals resulted in a sub- or even neofunctionalization of the genes. The chicken lacks the diversity of mammalian *CLCA* genes and the fully secreted CLCA 1/3 homologues seem to be dispensable for the chicken. In contrast, the expression of a glycosylated, a single TM domain containing CLCA protein like gCLCA1 or mammalian CLCA4 in the intestine suggests a taxon spanning, integral trait of CLCA homologues in this anatomical section.

## Supporting information

S1 File RT-qPCR of *gCLCA1* and *PGK1*. (DOCX)

S2 File In-detail protocols. (DOCX)

S3 File Testing of anti-gCLCA1 primary antibodies. (DOCX)

S4 File SNPs of the *gCLCA1* clone used in this study. (DOCX)

S5 File Detection of the N-terminal cleavage products of gCLCA1 in the cell supernatant using liquid chromatography-tandem mass spectrometry (LC-MS/MS). (DOCX)

S6 File EYFP- and HEK293 autofluorescence. (DOCX)

S7 File gCLCA1 protein expression at the apical brush border of bursal surface epithelium (red). (DOCX)

S1 Raw images (PDF)
