# Supplementary material for: Evolutionarily conserved properties of CLCA proteins 1, 3 and 4, as revealed by phylogenetic and biochemical studies in avian homologues
Source: PLoS One. 2022 Apr 13;17(4):e0266937. doi: 10.1371/journal.pone.0266937 (PMC9007345; doi:10.1371/journal.pone.0266937)
Supplement: S1 File — (DOCX) [file pone.0266937.s001.docx]

**S1 RT-qPCR raw data [Ct-values] of gCLCA1 and PGK1.**

| *Tissue* | *Animal 1* | | *Animal 2* | | *Animal 3* | |
| --- | --- | --- | --- | --- | --- | --- |
|  | *gCLCA1* | *PGK1* | *gCLCA1* | *PGK1* | *gCLCA1* | *PGK1* |
| Abdominal skin | 40.0 | 25.5 | 40.0 | 27.2 | 40.0 | 25.9 |
| Skin foot | 40.0 | 27.8 | 40.0 | 26.5 | 40.0 | 27.3 |
| Wattle | 40.0 | 27.1 | 40.0 | 26.5 | 37.4 | 27.2 |
| Feather follicle | 40.0 | 27.1 | 37.0 | 26.3 | 40.0 | 25.5 |
| Ball of the foot | 40.0 | 25.7 | 35.8 | 25.5 | 40.0 | 26.0 |
| Beak | 40.0 | 27.4 | 40.0 | 28.1 | 40.0 | 28.5 |
| Pharynx | 25.0 | 25.1 | 26.1 | 25.8 | 28.2 | 27.2 |
| Esophagus | 29.8 | 25.9 | 32.2 | 26.2 | 30.6 | 26.8 |
| Crop | 35.0 | 26.3 | 40.0 | 26.2 | 40.0 | 26.1 |
| Proventriculus | 36.6 | 27.2 | 40.0 | 27.4 | 40.0 | 29.7 |
| Gizzard | 38.2 | 27.0 | 35.5 | 27.2 | 35.8 | 28.3 |
| Duodenum | 24.5 | 25.7 | 26.4 | 26.6 | 26.4 | 27.5 |
| Jejunum | 20.0 | 25.6 | 21.1 | 25.9 | 27.4 | 28.6 |
| Ileum | 20.6 | 25.8 | 22.0 | 27.3 | 22.2 | 27.7 |
| Cecum | 23.3 | 25.6 | 24.6 | 27.0 | 24.3 | 28.1 |
| Rectum | 20.0 | 25.5 | 21.5 | 26.4 | 23.9 | 27.0 |
| Coprodeum | 22.2 | 25.4 | 22.0 | 26.3 | 24.6 | 27.9 |
| Proctodeum | 25.6 | 25.4 | 29.3 | 25.5 | 25.2 | 26.5 |
| Liver | 36.3 | 25.5 | 34.4 | 25.3 | 35.1 | 26.0 |
| Pancreas | 36.4 | 30.3 | 40.0 | 31.2 | 40.0 | 33.2 |
| Thyroid gland | 40.0 | 26.3 | 40.0 | 26.9 | 40.0 | 27.5 |
| Adrenal gland | 40.0 | 25.0 | 36.9 | 24.2 | 40.0 | 25.4 |
| Nose | 40.0 | 26.7 | 35.6 | 26.8 | 34.9 | 27.7 |
| Trachea | 40.0 | 26.2 | 35.3 | 26.4 | 40.0 | 24.3 |
| Tracheal bifurcation | 35.8 | 26.8 | 35.9 | 27.3 | 40.0 | 27.3 |
| Lung | 31.8 | 26.7 | 34.3 | 29.2 | 32.3 | 27.9 |
| Abdominal air sac | 40.0 | 28.5 | 40.0 | 30.6 | 40.0 | 25.9 |
| Heart | 40.0 | 25.6 | 38.3 | 25.2 | 40.0 | 26.7 |
| Vena cava | 40.0 | 25.5 | 40.0 | 30.4 | 40.0 | 29.6 |
| Aorta | 40.0 | 27.9 | 40.0 | 32.1 | 40.0 | 28.0 |
| Kidney | 35.1 | 24.2 | 35.1 | 24.1 | 40.0 | 25.1 |
| Ovary | 35.3 | 23.3 | 40.0 | 23.4 | 37.0 | 24.6 |
| Spleen | 36.2 | 26.3 | 40.0 | 28.2 | 36.1 | 29.0 |
| Thymus | 40.0 | 28.3 | 40.0 | 27.1 | 35.6 | 29.5 |
| Bursa of Fabricius | 27.4 | 24.5 | 32.3 | 24.7 | 34.1 | 26.2 |
| Bone marrow | 40.0 | 28.5 | 37.8 | 28.9 | 40.0 | 29.5 |
| Eye | 32.1 | 23.2 | 30.9 | 24.9 | 30.4 | 25.2 |
| Cerebrum | 36.2 | 25.9 | 36.3 | 24.9 | 34.2 | 25.1 |
| Cerebellum | 40.0 | 23.5 | 40.0 | 24.6 | 40.0 | 25.3 |
| Sciadic nerve | 35.5 | 31.0 | 36.0 | 28.1 | 40.0 | 26.1 |
| Pectoral muscle | 35.2 | 20.0 | 40.0 | 20.2 | 40.0 | 22.0 |
| Abdominal fat | 40.0 | 29.1 | 40.0 | 26.1 | 40.0 | 28.9 |
| Blood | 40.0 | 29.7 | 40.0 | 29.9 | 40.0 | 31.8 |
| Joint capsule | 40.0 | 29.6 | 40.0 | 28.1 | 40.0 | 29.6 |
| *Tissue* | *Animal 4* | | *Animal 5* | | *Animal 6* | |
|  | *gCLCA1* | *PGK1* | *gCLCA1* | *PGK1* | *gCLCA1* | *PGK1* |
| Testis | 40.0 | 23.4 | 40.0 | 25.4 | 40.0 | 27.6 |

**
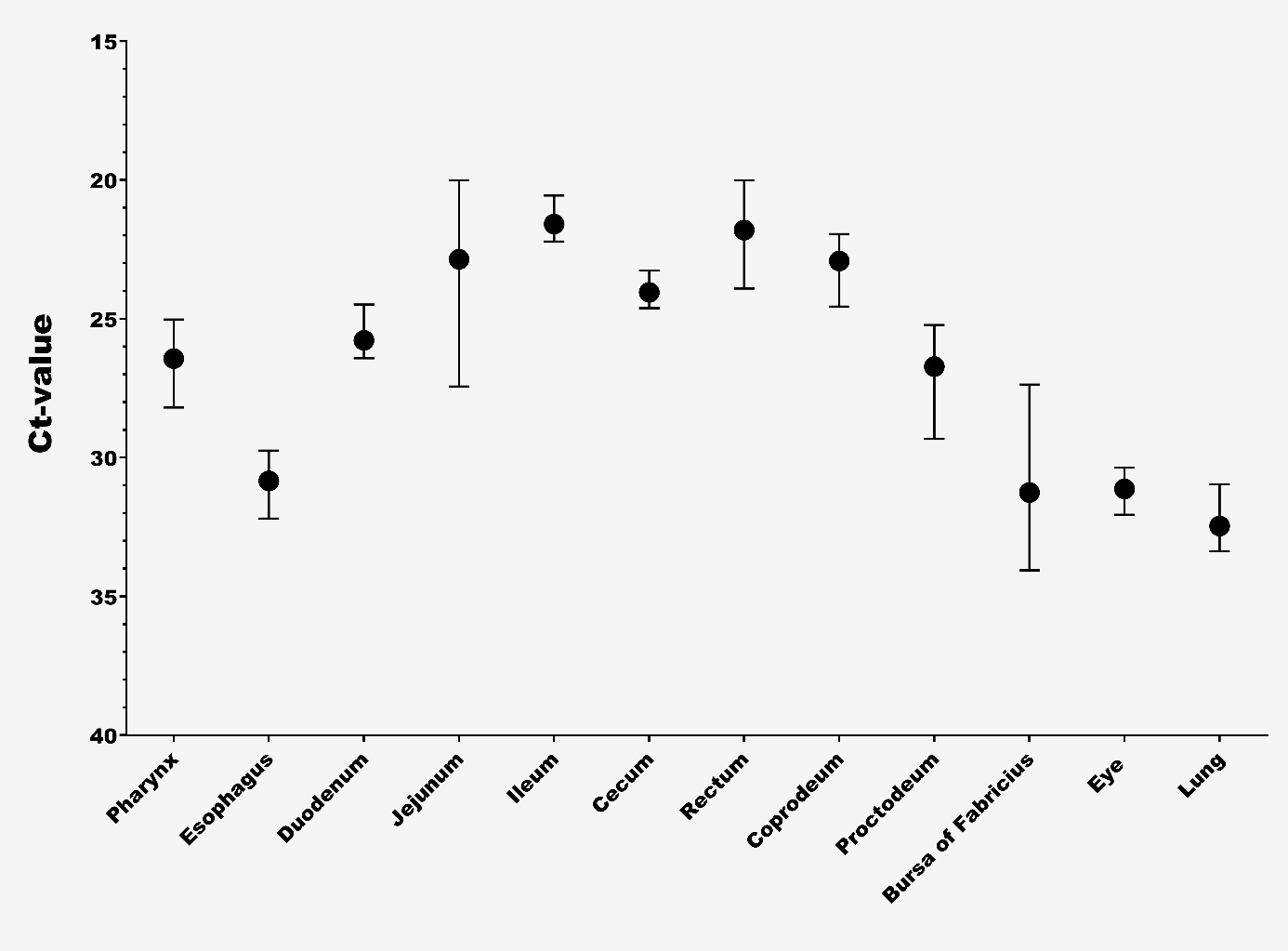
**

**Fig S1** **mRNA of *gCLCA1* was predominately expressed in the intestinal tract.** *gCLCA1* mRNA was detected in particular in different parts of the intestinal tract by RT-qPCR. Additionally, a weaker expression of *gCLCA1* was found in the upper alimentary tract, the Bursa of Fabricius, the eye, and the lung. Data are expressed as median (black dot) with range (error bars; n=3) and normalized to five ng total cDNA per reaction*.* The gene was considered to be expressed, when C_t_ –values below 35 were detected in at least two animals.
