# Supplementary material for: Evolutionarily conserved properties of CLCA proteins 1, 3 and 4, as revealed by phylogenetic and biochemical studies in avian homologues
Source: PLoS One. 2022 Apr 13;17(4):e0266937. doi: 10.1371/journal.pone.0266937 (PMC9007345; doi:10.1371/journal.pone.0266937)
Supplement: S2 File — (DOCX) [file pone.0266937.s002.docx]

**S2 In-detail protocols.**

Molecular cloning and sequencing of the *gCLCA1* gene

Total RNA was isolated from the proctodeum of animal 3 (Table S3) using the NucleoSpin RNA Mini Kit (Macherey-Nagel; Düren, Germany) according to the manufacturer´s protocol. RNA purity and concentration were determined using the Nanodrop-Spectrophotometer ND-1000 (PEQLAB, Erlangen, Germany). Five micrograms of RNA was reverse transcribed with the SuperScript III First-Strand Synthesis System (Invitrogen) using random hexamer and oligodT primers in equal parts for two hours at 55°C in the presence of 40 U RNaseOUT (Invitrogen). Specific primers were designed for the amplification of the *gCLCA1* ORF (Table S2-1).

| GenBank Gene ID | Oligonucleotide | Oligo sequence (5´-3´) | ORF size |
| --- | --- | --- | --- |
| 424523 | upstream | ACTAACGAGCTCATGTGGGGACAGGGACA | 2805 |
|  | downstream | TAACGCGGATCCAACATTCTGATTGCACGATCCTTTC |  |

Table S2-1. Primers for the amplification of the *gCLCA1* ORF.

The Q5-Hot Start High-Fidelity 2X Master Mix (New England Biolabs) was used according to the manufacturer´s protocol to amplify the *gCLCA1* ORF with PCR conditions as described (Table S2-2).

| Step | Temperature [°C] | Time [sec] | Repeats | Time increment [sec] |
| --- | --- | --- | --- | --- |
| Initial denaturation | 95.0 | 300 |  |  |
| Denaturation | 95.0 | 20 |  |  |
| Annealing | 63.6 | 20 |  |  |
| Extension | 68.0 | 240 | 10 |  |
| Denaturation | 95.0 | 20 |  |  |
| Annealing | 63.6 | 20 |  |  |
| Extension | 68.0 | 240 | 22 | 10 |
| Final Extension | 68.0 | 600 |  |  |

Table S2-2. PCR conditions for the amplification of the *gCLCA1* ORF.

The PCR products of eight reaction mixtures were pooled and separated via agarose gel electrophoresis with 1% TAE-low melting point agarose (Pharmacia LKB, Uppsala, Sweden). A band at the expected size of ~2800 bp was cut, purified using the NucleoSpin Gel and PCR Clean up Kit (Macherey-Nagel) and concentrated using a SpeedVac (RC1010, Thermo Fisher Scientific, Waltham, Massachusetts, USA) concentration centrifuge. The PCR products were then digested with SacI and BamHI (FastDigest restriction enzymes, Thermo Fisher Scientific). After a second purification and concentration step as described above, the digested PCR products were ligated with an analog linearized p*EYFP*-N1 vector (Clontech, Mountain View, California, USA) with a vector-insert-ratio of 1:17 using 2.5 U T4 Ligase (Thermo Fisher Scientific) over 12 hours. Electrocompetent *E. coli* (derived from OverExpress^TM^ strain C43, Lucigen, Middleton, Wisconsin, USA) were transformed by electroporation (Bio-Rad GenePulser, 1.8V) regenerated for 2 hours at 28°C in SOC-medium (0.5% Yeast extract, 2% Trypton, 10 mM NaCl, 2.5 mM KCl, 10 mM MgCl_2_, 10 mM MgSO_4_ 20 mM Glucose (all Carl Roth)) at 300 rpm. Subsequently, the cells were plated on a kanamycin-lysogeny broth (LB)-plate (50 mg/ml, Carl Roth GmbH, Karlsruhe, Germany), incubated at 28°C and picked after 12 hours. Single bacterial clones were screened using PCR. Positive clones were cultivated at 28°C and 300 rpm in a kanamycin (50 mg/ml, Carl Roth) containing LB-medium overnight and prepared using the NucleoBond Xtra EF Kit (Macherey-Nagel). The orientation of the inserted PCR product was verified by restriction analysis (Dra I, FastDigest restriction enzymes, Thermo Fisher Scientific). Three positive clones were subsequently sequenced using the primer walking method (Table S2-3) and compared to the reference sequence of the NCBI Genbank (XM_422360.6, red junglefowl (*Gallus gallus*)). The resulting clone was named *gCLCA1WT.*

| Location | Oligonucleotide | Oligo sequence (5´-3´) |
| --- | --- | --- |
| *CMV* Promoter | upstream | ATAGCGGTTTGACTCACGGG |
| *gCLCA1* | upstream | TGAAAATGCACGTGTTGAACCA |
| *gCLCA1* | upstream | GTGAAAGAAAGCGGGGCAATAA |
| *gCLCA1* | downstream | TGGTCTTTGCTTTCGAGCTG |
| *gCLCA1* | downstream | GTCAAACACACCCCACCGAA |
| *EYFP* | downstream | GGCGGACTTGAAGAAGTCGT |

Table S2-3. Primers used for sequencing of the *gCLCA1* ORF.

Generation of *gCLCA1E164Q, gCLCA1Nmabc1* and *gCLCA1Nmabc1E164Q* mutants

Two plasmids with a 789 bp long N-terminal gene fragment of *gCLCA1* were synthesized (GeneArt Gene Synthesis, Thermo Fisher Scientific) containing mutations as described below. The translation product of one synthesized plasmid coding for an internal recognition site for rabbit polyclonal anti-mCLCA1 (p3b, (53) recognizing the N-terminal cleavage product of *mCLCA1*) due to seven aa substitutions of the wild type gCLCA1 at positions 258, 259, 260, 263, 268, 271, 272 (KNTHNSEAPNQNKM 🡪 EKNHNQEAPNDQNQR). This synthesized plasmid was termed *Nmabc1* (Table S4). The second plasmid contained a mutation for an additional E 🡪 Q substitution at position 164 within the HExxH zinc-binding aa motif (80) and named *Nmabc1E164Q* (Table S2-4).

|  | Oligonucleotide sequence (5´-3´) | Position |
| --- | --- | --- |
| Nmabc1E164Q gCLCA1WT  Nmabc1E164Q gCLCA1WT | TCAGTGGGCCC  TGAGTGGGCTC  * ******* *  GAGAAGAACCACAACCAGGAGGCCCCCAACGATCAGAACCAGCGC  AAAAATACTCATAATTCTGAGGCTCCAAATATGCAGAATAAGATG  * ** * ** ** ***** ** ** ***** ** | 489-499  1393 - 1437 |
| Nmabc1  gCLCA1WT | GAGAAGAACCACAACCAGGAGGCCCCCAACGATCAGAACCAGCGC  AAAAATACTCATAATTCTGAGGCTCCAAATATGCAGAATAAGATG  * ** * ** ** ***** ** ** ***** ** | 1393 - 1437 |

Table S2-4. Nucleotide substitution used for generation of *E164Q* and *Nmabc1* mutations.

The *gCLCA1WT* and both of the synthesized plasmids were digested using ClaI and XmnI restriction enzymes (FastDigest, Thermo Fisher Scientific). In order to exclude an influence of *Nmabc1* on the E-Q mutation, a clone exclusively containing the E-Q mutant was generated. Therefore, *gCLCA1WT* and *Nmabc1E164Q* were digested with ClaI and PmII (FastDigest, Thermo Fisher Scientific) to obtain a 342 bp gene fragment and the corresponding plasmid backbone. The products were separated by agarose gel electrophoresis with 1% TAE-low melting point agarose (Pharmacia LKB) and purified with the NucleoSpin Gel and PCR Clean up kit (Macherey-Nagel). The backbones of *gCLCA1WT* were religated with the 789 bp respective 342 bp fragments using T4 Ligase (Thermo Fisher Scientific) according to the manufacturer´s recommendations and transformed by electroporation into *E. coli* as described above. Positive clones were verified by sequencing (Table S2-5). Clones containing only the E-Q mutations were named *gCLCA1E164Q*. Clones containing only the anti-mCLCA1 antibody recognition site were named *gCLCA1Nmabc1.* Clones containing both modifications were termed *gCLCA1Nmabc1E164Q*. All plasmids were prepared using the NucleoBond Xtra Midi EF-Kit (Macherey-Nagel) according to the manufacturer´s protocol.

| Location | Oligonucleotide | Oligo sequence (5´-3´) |
| --- | --- | --- |
| *gCLCA1* | upstream | GGGACAGGGACAAATAATGGG |
| *gCLCA1* | downstream | TGGTCTTTGCTTTCGAGCTG |

Table S2-5. Primers used for sequencing of the *gCLCA*1 mutants.

Tissue expression of *gCLCA1-*mRNA using RT-qPCR

Total RNA from approximately 100 mg of each tissue (Table S3) was isolated using the NucleoSpin RNA Mini Kit (Macherey-Nagel) according to the manufacturer´s protocol. 1 μg of total RNA was reverse transcribed using the Iscript cDNA Synthesis Kit (Bio-Rad, Watford, England) according to the manufacturer´s recommendations and the synthesized cDNA was diluted to a final concentration of 1 ng/μl. A specific *gCLCA1* detection system with upstream primers 5’-GCCCAAGGGAGAGATCAAAC-3’ and downstream primers 5’-CATCAGCCAATTCAGGTCTCG-3’ was designed to span exon–exon boundaries (exon 12 – 13, intron length: 1071 nucleotides (nt)). Each primer had a primer length between 20 and 21 nt and a guanine-cytosine content of 53 – 55%. The NCBI Primer-BLAST tool predicted a melting temperature of 58.65°C +/- 0.41 °C. The primer pair was designed to produce a single product with an amplicon length of 121 nucleotides. This single PCR product was indicated by a single peak in the melting curve analysis. The efficiency of amplification was determined to be 94.5% using the Maxima SYBR Green qPCR Master Mix (Thermo Fisher Scientific) under the following described (Table S2-6).

| Step | Temperature [°C] | Time [sec] | Repeats |
| --- | --- | --- | --- |
| Initial denaturation | 95.0 | 180 |  |
| Denaturation | 95.0 | 10 |  |
| Annealing + extension | 60 | 30 | 39 |

Table S2-6. PCR conditions for RT-qPCR of *gCLCA1.*

The PCR efficiency was calculated by the equation E = 10^(-1/slope)^-1 from an eight-fold serial dilution of 10^-1^ of the *gCLCA1WT* plasmid starting from 1 ng. The PCR product was sequenced to confirm amplification of the proper gene. To control for mRNA quality and efficacy of reverse transcription, a 167 bp product of the reference gene phosphoglycerate kinase (*PGK1*) was amplified from each sample using the primers 5’AAAGTTCAGGATAAGATCCAGCTG-3’ (upstream) and 5’-GCCATCAGGTCCTTGACAAT-3’ (downstream; (81)). RT-qPCR reactions were done in 96-well polypropylene plates (Bio-Rad) in a volume of 15 μl using a CX96 Real-Time PCR Detection System (BioRad). 5 ng cDNA and 6.7 moles of each primer were added to 10 μl Maxima SYBR Green qPCR Master Mix (Thermo Fisher Scientific). PCR reaction conditions for the tissue screening were as in Table VI. All reactions were run in doublets and the mean C_t_-values were used for further analysis if the standard deviation of the two C_t_-values was lower than 0.5. Nuclease-free water was used as no-template control.

Transient transfection of HEK293 cells

HEK293 (passage 22 – 35, ATCC, Manassas, Virginia, USA) cells were grown in DMEM (4.5 g/L glucose, 3.7 g/L NaHCO3, + pyruvate, + glutamate; Biochrom AG, Berlin, Germany) with 10% heat-inactivated FCS (Sigma-Aldrich), 1% HEPES (Biowest, Riverside, Missouri, USA) and 1% penicillin/streptomycin (Biowest), at 37°C. The supernatant was replaced by DMEM (Biochrom) containing 2% FCS (Sigma-Aldrich) when the cells were 80–90% confluent. The cells within each well were transfected with 2 μg of plasmid DNA (*gCLCA1WT*, *gCLCA1E164Q*, *gCLCA1Nmabc1*, *gCLCA1Nmabc1E164Q*, *mCLCA1* (Bothe et al. 2011), *mCLCA1E157Q* (Bothe et al. 2011), *mCLCA4a* (Bothe et al. 2012), *mCLCA4aE157Q* (Bothe et al. 2012),*EYFP*-mock orpcDNA3.1^+^-mock) mixed with 300 μl DMEM (Biochrom) and 8 μl PEI (PEI max 40, linear, 40 kDa, 1 mg/ml, Polysciences, Inc., Warrington, USA). 12 hours after transfection, the cells were washed with PBS (Biowest) 72 hours after transfection, the medium was removed, spun at 14,000 x g for one hour and concentrated using Vivaspin 2 protein concentrator spin columns with a 10 kDa molecular weight cutoff. The cells of each well were washed with prewarmed PBS (Biowest), lyzed with 500 μl RIPA-buffer (150 mM NaCl (Carl Roth), 25 mM Tris-HCl (Carl Roth) pH 7.4, 1 % Triton X-100 (Carl Roth), 0.5% Na-Deoxycholate (Carl Roth), 0.1% SDS (Carl Roth)) supplemented with a protease inhibitor cocktail (complete Mini, EDTA-free, Roche Diagnostics, Rotkreuz, Switzerland) according to the manufacturer´s recommendation and incubated on ice for 30 minutes. Crude cell lysates were spun at 14,000 x g for 5 minutes at 4°C to remove insoluble cell fragments and the pellet was discarded. The protein concentration of the supernatant and the cell lysate was determined using the Micro BCA Protein Assay Kit (Thermo scientific) according to the manufacturer´s protocol.

Immunoblotting

15 μg of cell lysates or concentrated cell supernatants were boiled in Laemmli buffer (6% SDS (Carl Roth), 30% glycerin (Carl Roth), 150 mM DTT (Carl Roth), 150 mM Tris-HCl (Carl Roth) pH 6.8, 0.02% bromophenol blue (Carl Roth)) for 10 minutes and separated by a Tris-glycine (Carl Roth) buffered SDS-PAGE using a 10% acrylamide (Carl Roth) gel. The proteins were blotted onto a PVDF-membrane (0.45 μm pore size, Carl Roth) and membranes were blocked with 5% non-fat milk (Carl Roth) in Tris-buffered saline with Tween 20 (TBS-T, 137 mM NaCl (Carl Roth), 2.7 mM KCl (Carl Roth), 24.8 mM Tris base (Carl Roth), 0.02% Tween-20 (Carl Roth)) for one hour. The membranes were then incubated with the antibodies gC1-N1, gC1-C1 at 5.0 μg/ml, 1.0 μg/ml and 0.5 μg/ml, or mouse monoclonal anti-YFP (affinity purified, abm, Vancouver, Canada) at 1:500, or rabbit polyclonal anti-mCLCA1 (53) at 1:500, or rabbit anti-mCLCA6 antibody (62), or mouse monoclonal anti beta-actin at 1:1,000 antibodies overnight at 4°C on a 3D shaker with 10 rpm. The membranes were washed three times with TBS-T for 15 minutes before incubation with horseradish peroxidase-conjugated secondary goat anti-rabbit antibodies (115-035-068, Jackson Immuno Research Laboratories Inc.) or goat anti-mouse (111-035-144, Jackson Immuno Research Laboratories, Inc.) at 1:8,000 for one hour at room temperature. The membranes were washed three times with TBS-T for 15 minutes before incubation with enhanced chemiluminescence (SuperSignal West Pico chemiluminescent substrate, Thermo Fisher Scientific). The protein bands were visualized via a ChemiDoc XRS gel documentation system with the Quantity One software package (version 4.6, BioRad, [Hercules, California, USA)](https://www.google.com/search?client=firefox-b-d&sxsrf=ALeKk01WO6m_EG4_pqLn8_6q5A01sWPTMg:1608039562874&q=Hercules+Contra+Costa+County,+CA,+Vereinigte+Staaten&stick=H4sIAAAAAAAAAOPgE-LSz9U3MC4wzDVPUeIAsQsrCwu1tLKTrfTzi9IT8zKrEksy8_NQOFYZqYkphaWJRSWpRcWLWE08UouSS3NSixWc8_NKihKBVHEJiCzNK6nUUXB21FEISy1KzczLTC9JVQguSUwsSc3bwcoIAEusOFR-AAAA&sa=X&ved=2ahUKEwiB3LeXjtDtAhWi2-AKHTveBmsQmxMoATARegQIHBAD). No anti-gCLCA1 antibodies detected any blotted gCLCA1 protein.

Immunocytochemistry

HEK293 cells (passage 20 – 25, ATCC) were grown as described above and seeded in 8-well tissue culture chambers on PCA slides (Sarstedt, Nümbrecht, Germany, density: 3 x 10^4^ cells per well). The 80–90% confluent cells were transfected using 250 ng of either *gCLCA1WT* or *EYFP-*mock plasmid DNA mixed with 37.5 μl serum-free medium and l μl PEI (PEI max 40, Polysciences). 72 hours after transfection, the cells were fixed with icecold methanol (Carl Roth) for 5 minutes followed by a paraformaldehyde (4% in PBS, pH 7.4, Alfa Aesar, Thermo Fisher Scientific) fixation for 10 minutes at room temperature. Each well was washed three times with 300 μl PBS (Biowest), permeabilized with 0.1 % Triton X-100 (Carl Roth) in PBS (Biowest) for 10 minutes and blocked with 300 μl blocking buffer (PBS (Biowest) containing 10% GS (Sigma-Aldrich) and 0.05% Tween 20 (AppliChem) per well for 30 minutes. The fixed cells transfected with *gCLCA1WT* or *EYFP-*mock were incubated at 4°C overnight with the antibodies gC1-N1 or gC1-C1, which were diluted to a final concentration of 1 μg/ml. To control for specificity, transfected cells were incubated with an irrelevant affinity-purified antibody (anti-pig CFTR (83)) with a final concentration of 1 μg/ml. Additionally, gC1-N1 and gC1-C1 at 1 μg/ml were pre-absorbed with 50 μg/ml of the specific peptides (gCN1: KKNSTYSRLKTESY, gCC1:ASVPSDDEGNTSDG) or an irrelevant peptide (LLPHRNSSKQRSRS (83)) on a 3D shaker with 12 rpm for one hour at room temperature. *gCLCA1WT* or *EYFP-*mock transfected cells were then incubated with the pre-absorbed antibodies at 4°C overnight. After incubation with primary antibodies, the cells were washed three times with 300 μl PBS (Biowest) and incubated with Alexa Fluor 568-conjugated goat anti-rabbit (AB_143157, Invitrogen), or goat anti-mouse secondary antibodies (AB_2534072, Invitrogen) at 1:200 for one hour at room temperature. The cells were washed three times with 300 μl PBS (Biowest), counterstained with DAPI nuclear stain (ROTIMount FluorCare DAPI, Carl Roth) and coverslipped. The slides were analyzed using an Olympus BX41 fluorescence microscope (Olympus, Shinjuku, Tokyo, Japan) with CellSens 1.18 (Olympus, Shinjuku, Tokyo, Japan) software. In contrast to the gC1-C1 antibody, the gC1-N1 antibody did not detect any gCLCA1 protein.

Tissue expression of gCLCA1 using immunofluorescence and immunohistochemistry

Formalin-fixed paraffin-embedded tissues, which were positive for gCLCA1 expression on the mRNA-level, were cut at 1 μm thickness and mounted on adhesive glass slides (STAR FROST ADHESIVE slides, Knittel glass, Braunschweig, Germany). The slides were dewaxed two times in xylene for 10 minutes and rehydrated in doublets with decreasing concentrations (100%, 95%, 70%) of ethanol (Carl Roth) for 3 minutes each. Only for DAB (Merck, Darmstadt, Germany) and alcian blue (AB)-counterstained sections, endogenous peroxidase was blocked by incubating the slides with 0.5% H_2_O_2_ (Carl Roth) in methanol (Carl Roth) for 20 minutes. The antigen was retrieved for slides incubated with the gC1-C1 antibody using 1 mg/ml recombinant protease from *Streptomyces griseus* (PanReac Applichem, Darmstadt, Germany) for 13 minutes at 37°C and for slides incubated with the anti-villin antibody microwave heating (600 W) in 10 mM citric acid, pH 6.0, containing 0.05% Triton X-100 for 15 minutes. For immunohistochemistry, slides were incubated after epitope retrieval with 10% Roti-Immunoblock (Carl Roth) and 20% GS (Sigma-Aldrich) in PBS (Biowest) for 30 minutes followed by an incubation with gC1-C1 with a final dilution of 1 μg/ml or rabbit monoclonal anti-villin (ab130751, Abcam, Cambridge, United Kingdom) at 1:400 or or with an irrelevant, affinity-purified rabbit antibody (anti-pig CFTR (83)) at 4°C overnight. The sections were washed three times with washing buffer and incubated for one hour with a biotinylated goat anti-rabbit secondary antibody (BA-1000, Vector Laboratories) diluted 1:200. The DAB (Merck) staining was achieved by incubating the sections with freshly prepared ABC solution (VECTASTAIN Original ABC Kit, Vector Laboratories), followed by repeated washes with washing buffer and exposure to DAB (Merck) for 8 minutes. The sections were counterstained in 1% AB (Carl Roth) in HCl (Carl Roth), pH 1.0 for 30 minutes followed by an incubation in 0.1% nuclear fast red-aluminium sulfate (Sigma-Aldrich) for 7 minutes, dehydration with increasing concentrations of ethanol (70%, 95%, 100%, Carl Roth), clearing in xylene (Carl Roth) and coverslipping. The slides were analyzed using an Olympus BX41 fluorescence microscope (Olympus, Shinjuku, Tokyo, Japan) with CellSens 1.18 (Olympus, Shinjuku, Tokyo, Japan) software.

Sections, which were analyzed via immunofluorescence were washed with washing buffer (PBS (Biowest) with 0.05% Triton X-100 (Carl Roth)) followed by permeabilization with 0.1 % Triton X-100 (Carl Roth) in PBS (Biowest) for 10 minutes. The slides were washed three times with PBS (Biowest) and incubated with blocking buffer (10% GS and 0.05% Tween 20 in PBS (Biowest)) for 30 minutes. The sections were incubated with gC1-C1 at 1 μg/ml or with an irrelevant, affinity-purified rabbit antibody (anti-pig CFTR (83)) with a final concentration of 1 μg/ml at 4°C overnight. The sections were washed three times with washing buffer and incubated with Alexa fluor 568-conjugated goat anti-rabbit (AB_143157, Invitrogen) secondary antibodies at 1:200 for one hour at room temperature. The sections were then washed three times with washing buffer, counterstained with DAPI (ROTIMount FluorCare DAPI, Carl Roth) and coverslipped.
