# Supplementary material for: Evolutionarily conserved properties of CLCA proteins 1, 3 and 4, as revealed by phylogenetic and biochemical studies in avian homologues
Source: PLoS One. 2022 Apr 13;17(4):e0266937. doi: 10.1371/journal.pone.0266937 (PMC9007345; doi:10.1371/journal.pone.0266937)
Supplement: S3 File — (DOCX) [file pone.0266937.s003.docx]

**
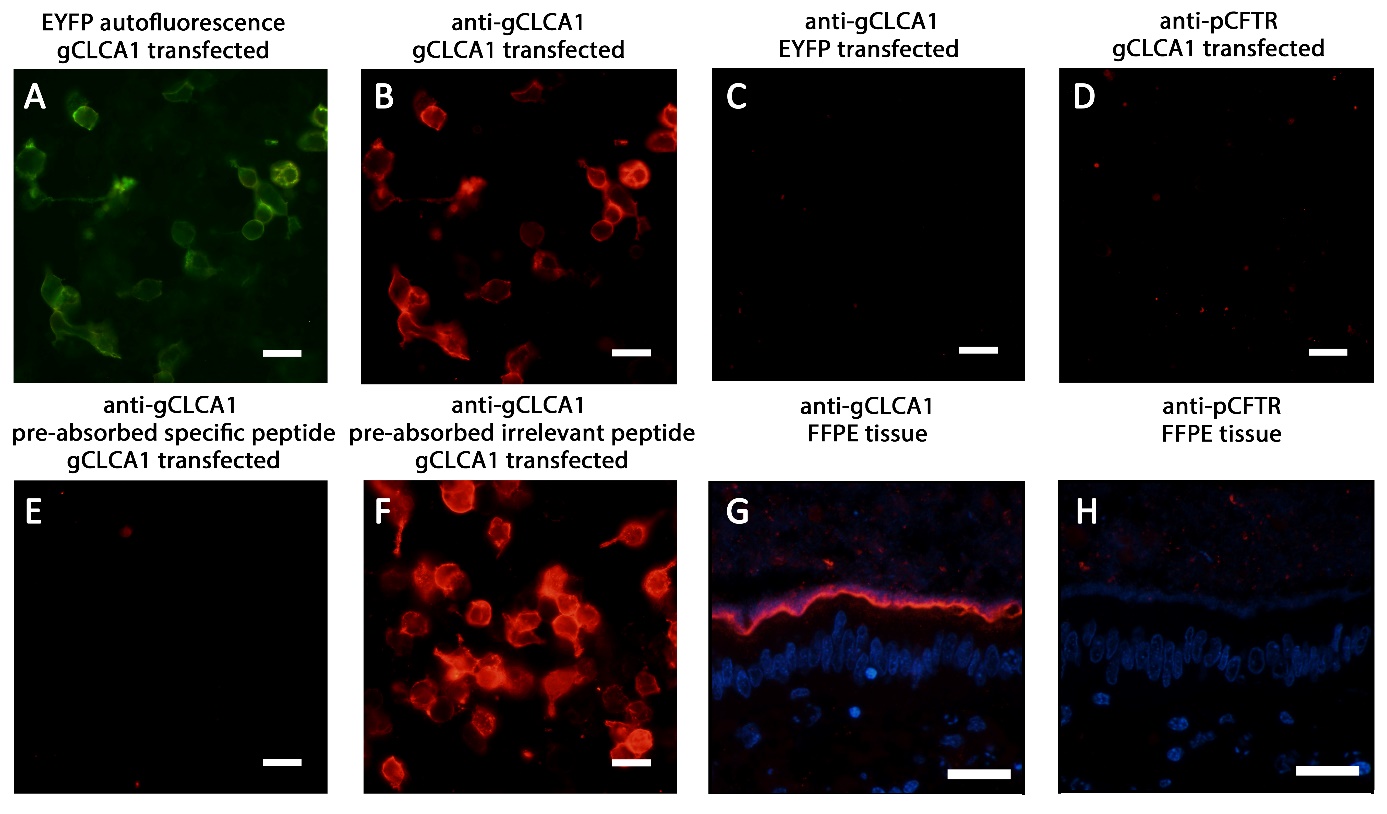
**

**Fig S3-1** **Testing of gC1-C1 primary antibody for specificity.**

EYFP auto- and immunofluorescence of HEK293 cells transiently transfected with the *gCLCA1WT* plasmid (A, B, D, E, F) or *EYFP*-mock plasmid (EYFP, C) plasmids. (B) The signal (red) detected with the gC1-C1 primary antibody was virtually identical to the autofluorescence signal (green) in A. No specific signals were detected after incubation of mock transfected cells with the antibody (C) or when *gCLCA1WT* transfected cells were incubated with an irrelevant antibody (anti-pCFTR, Plog et al. 2010) (D). The incubation of *gCLCA1WT* transfected cells with the pre-absorbed gC1-C1 antibody using the specific peptide for immunization did not detect any gCLCA1 protein (E). In contrast, the pre-absorption of the gC1-C1 antibody with an irrelevant peptide did not reduce the signal intensity (F). After incubation of FFPE sections from chicken rectum with the gC1-C1 antibody, a strong red signal was identified at the apical surface of non-goblet cell enterocytes (G). This signal was not detected when identical sections were incubated with an irrelevant (anti-pCFTR) primary antibody. Alexa fluor 568-conjugated secondary antibodies (B-H) and DAPI counterstain (blue, G-H). Bars indicate 20 μm. Exposure times were 1 second for A, 190 milliseconds (ms) for B-H (Alexa fluor 488) and 50 ms for G-H (DAPI).


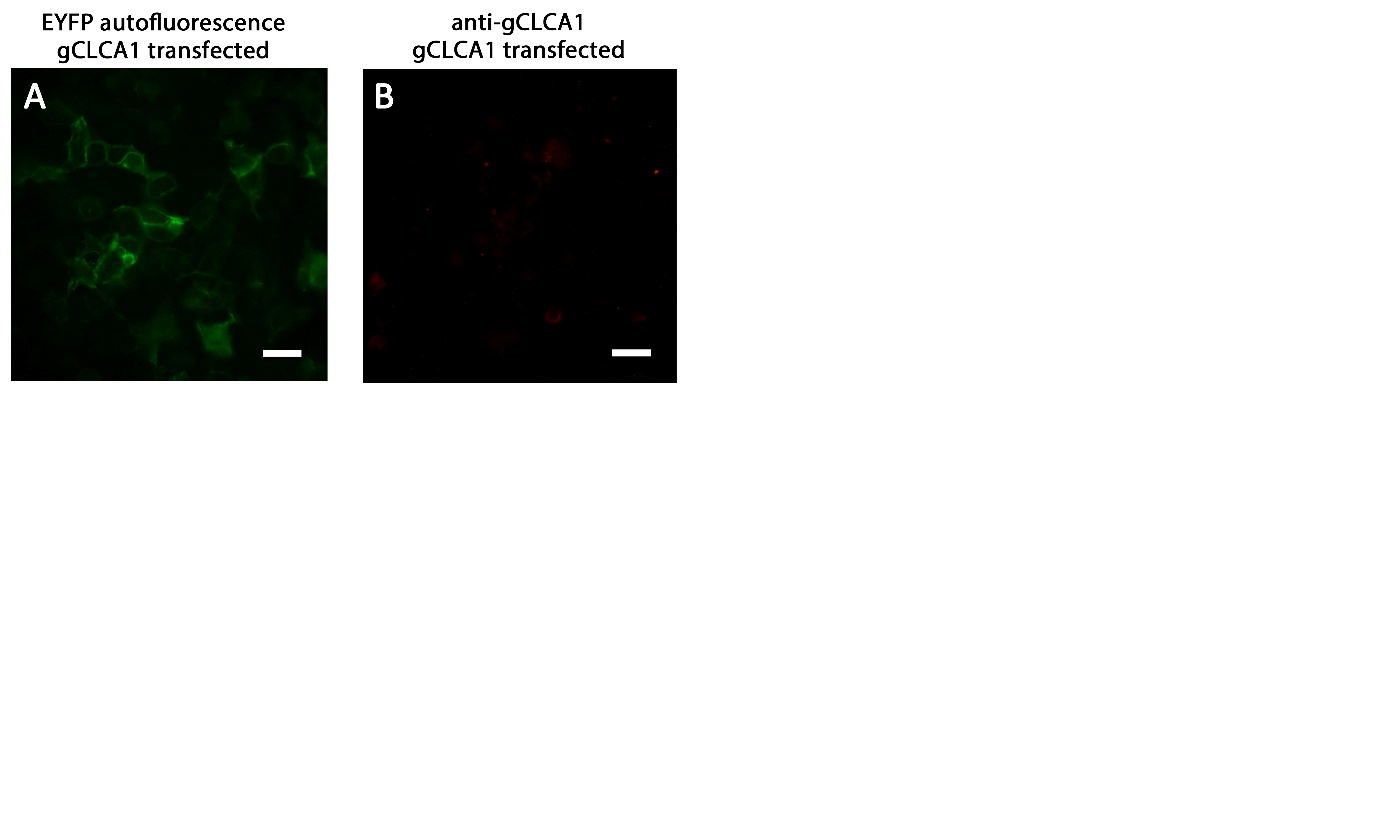


**Fig S3-2** **Testing of gC1-N1 primary antibody for immunofluorescence application.**

Autofluorescence of the EYFP tagged gCLCA1 in HEK293 cells transiently transfected with the *gCLCA1WT* plasmid (A). No gCLCA1 specific signals were detected after incubation of *gCLCA1WT* transfected cells with 1 μg/ml of the gC1-N1 antibody (B). This shows that this antibody is not applicable for immunofluorescence application. Bars indicate 20 μm. Exposure times were 1 second for A and 190 ms for B.


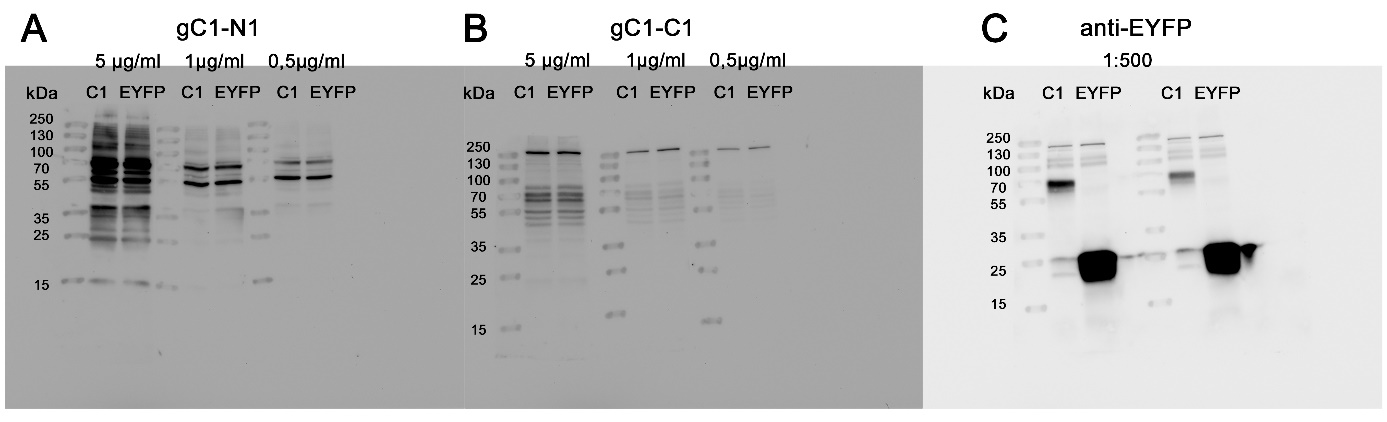


**Fig S3-3** **Testing of gC1-N1 and gC1-N1 primary antibodies for immunoblot application.**

Immunoblotting of lysates from HEK293 cells transfected with the *gCLCA1WT* (C1) or *EYFP*-mock plasmid (EYFP, A-C). No gCLCA1 specific bands were detectable using the antibodies gC1-N1 or gC1-C1 in three different concentrations (A, B). This shows that these antibodies are not applicable for immunoblot application. To control for expression of gCLCA1 or EYFP, cell lysates were analyzed using an anti-EYFP antibody (C).
