# Supplementary material for: Evolutionarily conserved properties of CLCA proteins 1, 3 and 4, as revealed by phylogenetic and biochemical studies in avian homologues
Source: PLoS One. 2022 Apr 13;17(4):e0266937. doi: 10.1371/journal.pone.0266937 (PMC9007345; doi:10.1371/journal.pone.0266937)
Supplement: S4 File — (DOCX) [file pone.0266937.s004.docx]

| **Non synonymus single nucleotide polymorphisms** | | **Synonymus single nucleotide polymorphisms** |
| --- | --- | --- |
| **mRNA level** | **Protein level** | **mRNA level** |
| c.448A>T | p.150M>L | c.1116T>C |
| c.533A>T | p.178N>S | c.1155A>C |
| c.1352C>A | p.415A>E | c.1605A>G |
| c.1997A>G | p.666K>R | c.2355G>A |

**S4** **SNPs of the *gCLCA1* clone used in this study.**
