# Supplementary material for: Evolutionarily conserved properties of CLCA proteins 1, 3 and 4, as revealed by phylogenetic and biochemical studies in avian homologues
Source: PLoS One. 2022 Apr 13;17(4):e0266937. doi: 10.1371/journal.pone.0266937 (PMC9007345; doi:10.1371/journal.pone.0266937)
Supplement: S5 File — (DOCX) [file pone.0266937.s005.docx]

**S5 Detection of the N-terminal cleavage products of gCLCA1 in the cell supernatant using liquid chromatography-tandem mass spectrometry (LC-MS/MS).**

In order to control for qualitative impacts of the Nmabc1 mutation on the secretion of the N-terminal cleavage product of gCLCA1, in a single experiment, the cell culture supernatant of *gCLCA1WT*-transfected cells was screened for the presence of the gCLCA1 N-terminus using LC-MS/MS. 25 μg of cell culture supernatant was obtained from *gCLCA1WT*-transfected cells as described above. The samples were prepared for SDS-PAGE as described above and separated in a 12.5% acrylamide (Carl Roth) gel. Gel bands around the expected size of the N-terminus (80-130 kDa) were excised separately and subjected to trypsin in-gel digestion as described previously (1). Peptides were dissolved in 10 μl of 0.1% (v/v) TFA, 5% (v/v) acetonitrile and 2 μl were analyzed on a Ultimate 3000 reversed-phase capillary nano liquid chromatography system connected to an Orbitrap Velos mass spectrometer (Thermo Scientific). Samples were desalted on a trap column (PepMap100 C18, 3 μm, 100 Å, 75 μmi.d.×2 cm; Thermo Scientific) using a mobile phase of 0.05% TFA, 2% acetonitrile in water. After switching the trap column inline, LC separations were performed on a capillary column (Acclaim PepMap100 C18, 2 μm, 100 Å, 75 μm i.d.×25 cm, Thermo Scientific) at an eluent flow rate of 300 nl/min. Mobile phase A contained 0.1% formic acid in water, mobile phase B contained 0.1% formic acid in acetonitrile. The column was pre-equilibrated with 3% mobile phase B followed by an increase to 50% mobile phase B in 50 min. Mass spectra were acquired in a data-dependent mode, utilizing a single MS survey scan (m/z 350–1500) with a resolution of 60,000 in the Orbitrap, and MS/MS scans of the 20 most intense precursor ions in the linear trap quadrupole. The dynamic exclusion time was set to 60s and automatic gain control was set to 1 x 10^6^ and 5,000 for Orbitrap-MS and LTQ-MS/MS scans, respectively.

Data processing and analysis

Identification of proteins was performed using the Mascot Daemon and Mascot Server version 2.5.0 (Matrix Science). Raw data from all gel bands were merged and searched against the human Uniprot reference proteome (75,776 entries, release October 18, 2020) extended by the sequences of the transfected constructs (*gCLCA1WT*). A maximum of two missed cleavages was allowed and the mass tolerance of precursor and sequence ions was set to 20 ppm and 0.35 Da, respectively. Carbamidomethyl (C) was set as fixed modification. Oxidation (M) and acetylation (protein N-terminus) were set as variable modifications. A significance threshold of 0.05 was used based on decoy database searches and a peptide ion score cut-off of 20 was applied.

Results

Exclusively tryptic peptides belonging to the N-terminal cleavage fragments of gCLCA1WT were detected in the supernatant of gCLCA1WT transfected cells (Table S4-1, Fig S4-1).

| Residue number in gCLCA1-EYFP | Peptide sequence  (dots indicate cleavage site) | Observed  m/z | Experimental  mass [Da] | Theoretical  mass [Da] | Mass  deviation [ppm] | Number of missed cleavage sites | Peptide score (Mascot) | Expect value (Mascot) |
| --- | --- | --- | --- | --- | --- | --- | --- | --- |
| 065 – 075 | K.DASNYLFEATK.H | 629.7992 | 1257.5838 | 1257.5877 | -3.09 | 0 | 71 | 2.0E-07 |
| 100 – 107 | R.LKTESYNK.A | 491.7635 | 981.5124 | 981.5131 | -0.66 | 1 | 16 | 2.9E-02 |
| 108 – 118 | K.ADVIIADPYLK.Y | 609.3395 | 1216.6644 | 1216.6703 | -4.84 | 0 | 44 | 6.5E-04 |
| 352 – 364 | R.VGIVTFESSAYEK.S | 715.3628 | 1428.7111 | 1428.7137 | -1.8 | 0 | 78 | 5.9E-07 |
| 365 – 376 | K.SPLLQITSVATR.Q | 643.3760 | 1284.7375 | 1284.7401 | -2.02 | 0 | 70 | 4.6E-07 |
| 379 – 392 | R.LVQNLPTTAGGGTK.I | 678.8756 | 1355.7367 | 1355.7409 | -3.06 | 0 | 35 | 6.1E-04 |
| 477 – 501 | K.LVEAFSEITTGSGDISEQSIQLESK.D | 890.1116 | 2667.3129 | 2667.3127 | 0.09 | 0 | 29 | 2.0E-03 |
| 534 – 541 | K.KQPLFFVR.D | 517.8102 | 1033.6059 | 1033.6073 | -1.33 | 1 | 18 | 3.9E-02 |
| 545 – 561 | K.GKEYGSSDFTIDSSNLK.T | 616.6257 | 1846.8553 | 1846.8585 | -1.73 | 1 | 31 | 1.5E-03 |
| 667 – 673 | K.NDGIYSR.Y | 412.6981 | 823.3817 | 823.3824 | -0.91 | 0 | 28 | 3.8E-03 |
| 674 – 683 | R.YFTSLSGDGR.Y | 551.7615 | 1101.5084 | 1101.5091 | -0.59 | 0 | 51 | 8.0E-05 |

**Table S5. Tryptic peptides specific for gCLCA1 detected in the supernatant of gCLCA1WT transfected HEK293 cells using LC-MS/MS.**

These data were obtained from a single experiment.


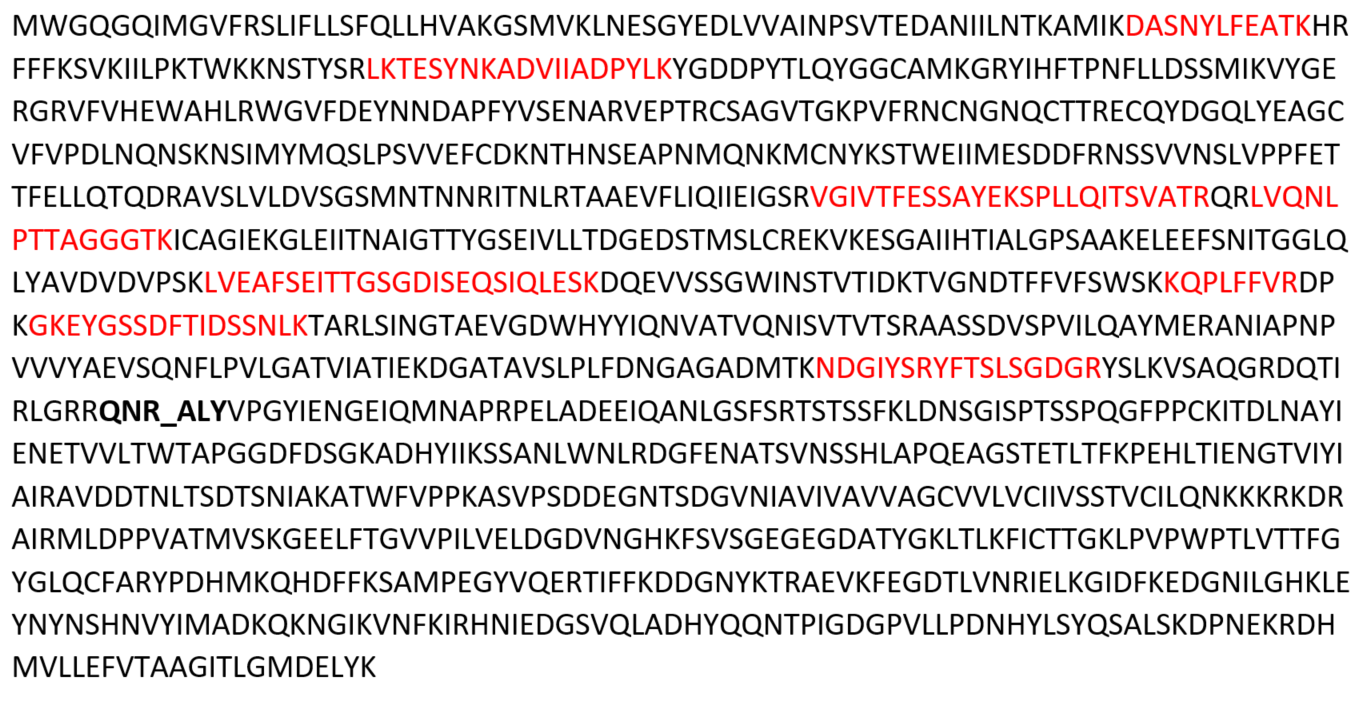


**Fig S5.** **Tryptic peptides specific for gCLCA1 detected in the supernatant of gCLCA1WT transfected HEK293 cells using LC-MS/MS projected on the *gCLCA1WT* sequence.** Only peptides belonging to the N-terminal cleavage fragment were detected. Red letters = detected peptides, bold letters (QNR_ALY) = putative cleavage site. These data were obtained from a single experiment

References

1. Shevchenko A, Wilm M, Vorm O, Mann M. Mass spectrometric sequencing of proteins from silver-stained polyacrylamide gels. Analytical chemistry. 1996;68(5):850-8.
