# Supplementary material for: Evolutionarily conserved properties of CLCA proteins 1, 3 and 4, as revealed by phylogenetic and biochemical studies in avian homologues
Source: PLoS One. 2022 Apr 13;17(4):e0266937. doi: 10.1371/journal.pone.0266937 (PMC9007345; doi:10.1371/journal.pone.0266937)
Supplement: S6 File — (DOCX) [file pone.0266937.s006.docx]

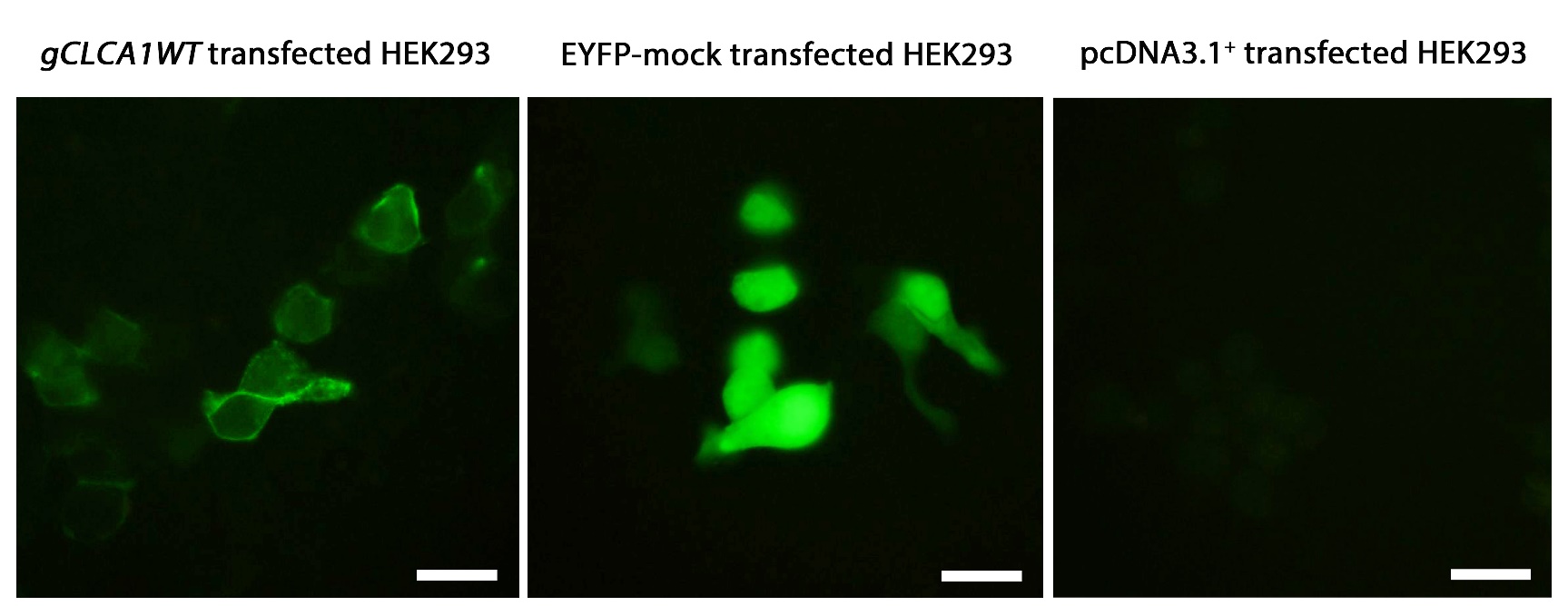


**Fig S6** **specifity control of the EYFP autofluorescence signal.** A green signal of the C-terminal EYFP-tag was detected along the plasma membrane of cells transfected with the *gCLCA1WT* plasmid (left panel). This is in contrast to the diffuse green, cytoplasmic autofluorescent EYFP signal as detected in cells transfected with the EYFP-mock plasmid (central panel) and the sparse autofluorescent background signal detected in HEK293 cells transfected with the pcDNA3.1+ plasmid that lacks an autofluorescent tag (right panel). White bars represent 20 μm.
