# Supplementary material for: Evolutionarily conserved properties of CLCA proteins 1, 3 and 4, as revealed by phylogenetic and biochemical studies in avian homologues
Source: PLoS One. 2022 Apr 13;17(4):e0266937. doi: 10.1371/journal.pone.0266937 (PMC9007345; doi:10.1371/journal.pone.0266937)
Supplement: S7 File — (DOCX) [file pone.0266937.s007.docx]

**
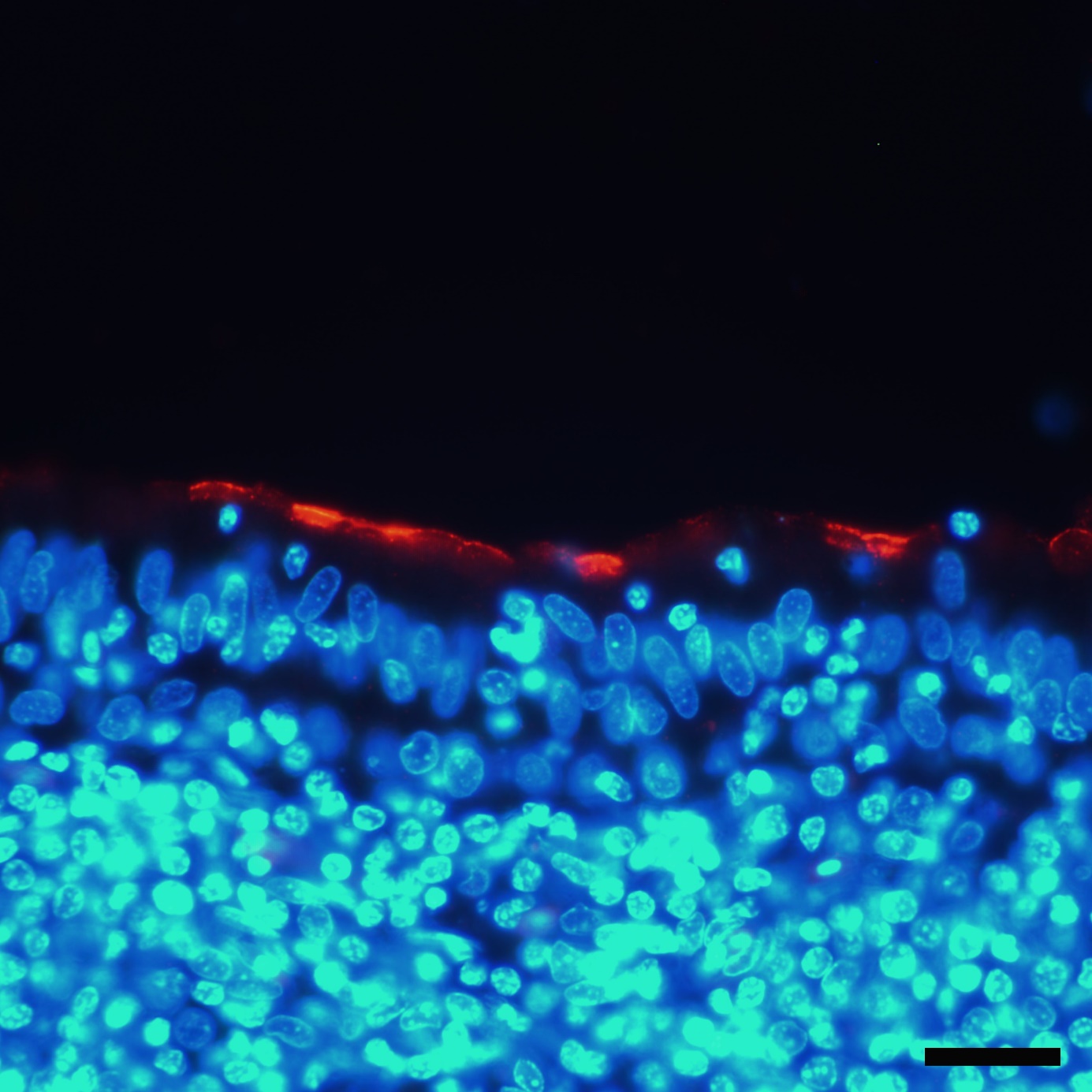
**

**Fig S7 gCLCA1 protein expression at the apical brush border of bursal surface epithelium (red).**

Immunofluorescence with the gC1-C1 (anti-gCLCA1 C-terminal) antibody. Alexa fluor 568-conjugated secondary antibodies and DAPI counterstain (blue). Bar indicates 20 μm.
