# Supplementary material for: Evolutionarily conserved properties of CLCA proteins 1, 3 and 4, as revealed by phylogenetic and biochemical studies in avian homologues
Source: PLoS One. 2022 Apr 13;17(4):e0266937. doi: 10.1371/journal.pone.0266937 (PMC9007345; doi:10.1371/journal.pone.0266937)

S6 original blots

Figure 4: Cell lysates from cells transfected with gCLCA1, gCLCA1EQ and EYFP-mock plasmids

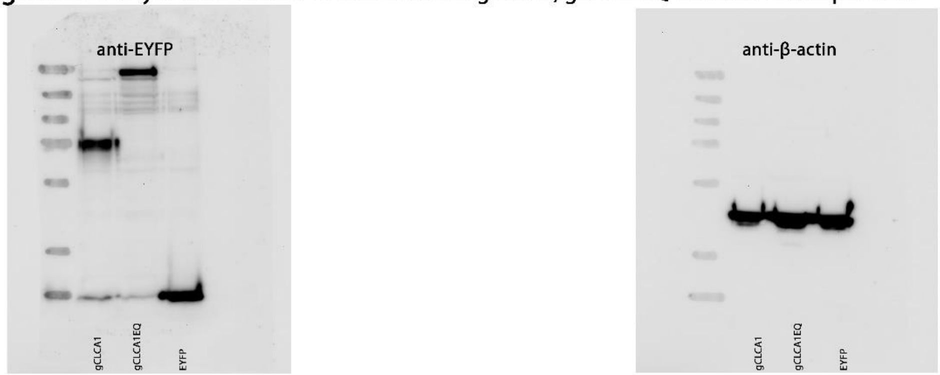

Figure 5: Cell lysates from cells transfected with gCLCA1, gCLCA1EQ, mCLCA1, mCLCA1EQ, mCLCA4a, mCLCA4aEQ, pcDNA-mock and EYFP-mock plasmids

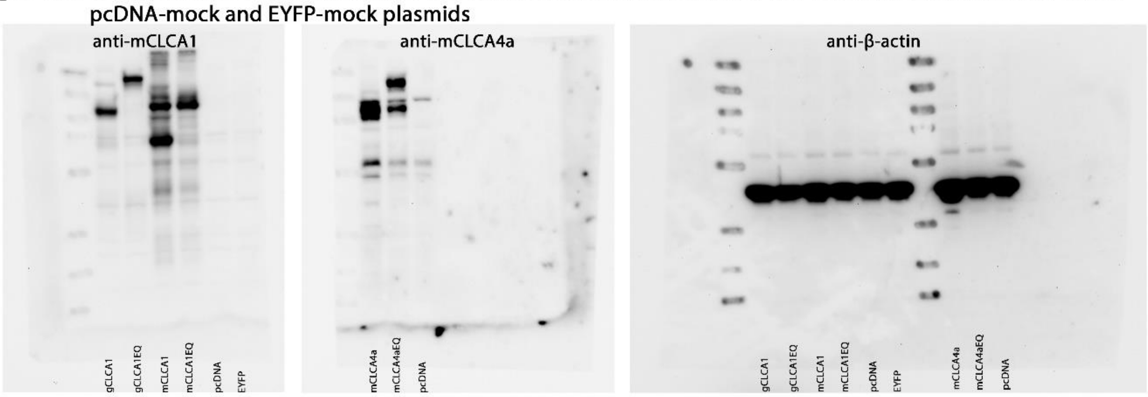

Figure 6: Cell lysate (L) and supernatant (S) from cells transfected with gCLCA1Nmabc1 and EYFP-mock plasmids

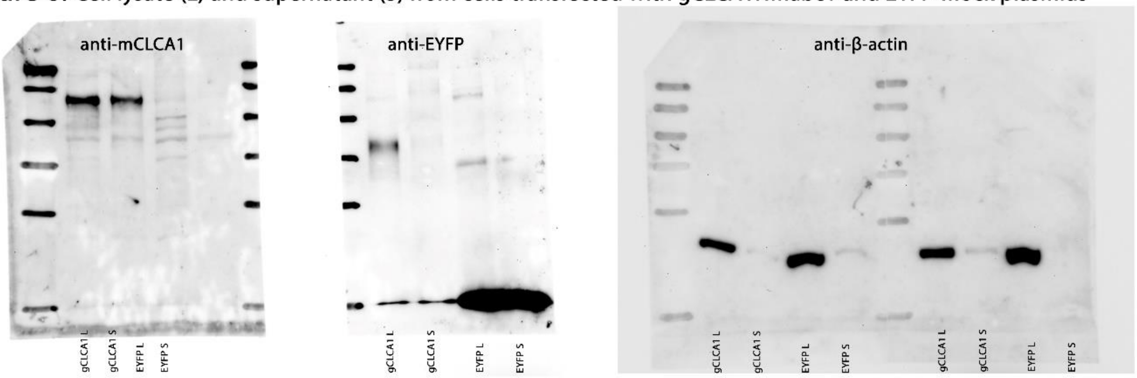

Figure 7: Cell lysate from cells transfected with gCLCA1Nmabc1 and EYFP-mock plasmids treated with endo H (H) or PNGase F (F) or left untreated (U)

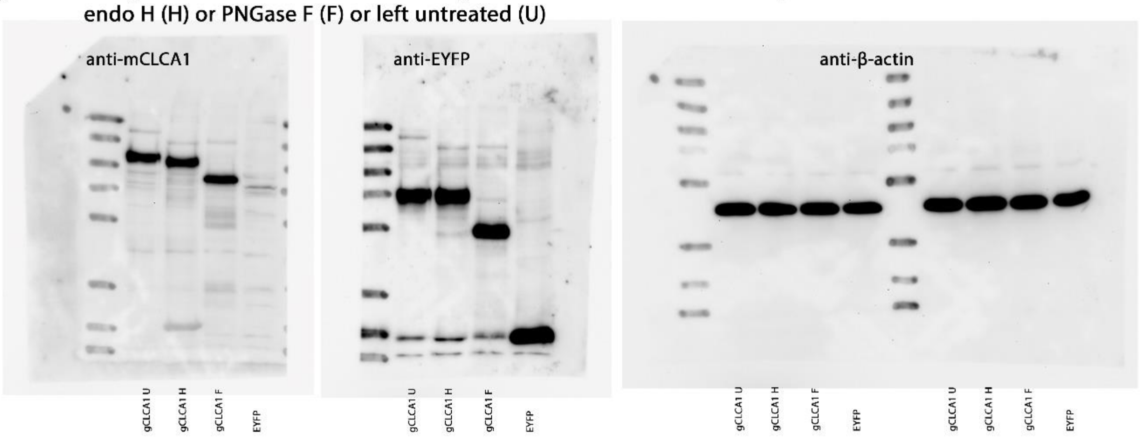

Supplement: S1 Raw images — (PDF) [file pone.0266937.s008.pdf]
